# Multiscale Hy3S: Hybrid stochastic simulation for supercomputers

**DOI:** 10.1186/1471-2105-7-93

**Published:** 2006-02-24

**Authors:** Howard Salis, Vassilios Sotiropoulos, Yiannis N Kaznessis

**Affiliations:** 1 Dept of Chemical Engineering & Materials Science and the Digital Technology Center, University of Minnesota, Minneapolis, Minnesota, 55455, USA

## Abstract

**Background:**

Stochastic simulation has become a useful tool to both study natural biological systems and design new synthetic ones. By capturing the intrinsic molecular fluctuations of "small" systems, these simulations produce a more accurate picture of single cell dynamics, including interesting phenomena missed by deterministic methods, such as noise-induced oscillations and transitions between stable states. However, the computational cost of the original stochastic simulation algorithm can be high, motivating the use of hybrid stochastic methods. Hybrid stochastic methods partition the system into multiple subsets and describe each subset as a different representation, such as a jump Markov, Poisson, continuous Markov, or deterministic process. By applying valid approximations and self-consistently merging disparate descriptions, a method can be considerably faster, while retaining accuracy. In this paper, we describe Hy3S, a collection of multiscale simulation programs.

**Results:**

Building on our previous work on developing novel hybrid stochastic algorithms, we have created the Hy3S software package to enable scientists and engineers to both study and design *extremely large *well-mixed biological systems with many *thousands *of reactions and chemical species. We have added adaptive stochastic numerical integrators to permit the robust simulation of dynamically *stiff *biological systems. In addition, Hy3S has many useful features, including embarrassingly parallelized simulations with MPI; special discrete events, such as transcriptional and translation elongation and cell division; mid-simulation perturbations in both the number of molecules of species and reaction kinetic parameters; combinatorial variation of both initial conditions and kinetic parameters to enable sensitivity analysis; use of NetCDF optimized binary format to quickly read and write large datasets; and a simple graphical user interface, written in Matlab, to help users create biological systems and analyze data. We demonstrate the accuracy and efficiency of Hy3S with examples, including a large-scale system benchmark and a complex bistable biochemical network with positive feedback. The software itself is open-sourced under the GPL license and is modular, allowing users to modify it for their own purposes.

**Conclusion:**

Hy3S is a powerful suite of simulation programs for simulating the stochastic dynamics of networks of biochemical reactions. Its first public version enables computational biologists to more efficiently investigate the dynamics of realistic biological systems.

## Background

The stochastic simulation of chemical and biochemical reaction networks, also known as kinetic Monte Carlo, has been successfully used to accurately predict the intracellular dynamics of biological organisms [1-6], including behavior that is not captured by deterministic methods, such as noise-induced oscillations [7], transitions between stable states [8], population heterogeneity [9], stochastic focusing and resonance [10], and smoothing of critical bifurcation points [11]. Because these probabilistic effects alter both the quantitative and qualitative dynamics of a system, the use of stochastic methods is critical to understanding natural biological systems and designing synthetic ones. The original stochastic simulation algorithm [12], or its improved variants [13,14], have been primarily used because they produce *exact *realizations of a jump Markov process with discrete states. This mathematical representation capably describes how the number of molecules of each unique chemical species in a well-mixed single cell changes over time, including the fluctuations arising from thermal noise. The same mathematical description can also be extended to reaction-diffusion systems describing heterogeneous intracellular dynamics [15].

However, since these methods individually execute reaction or diffusion events, the computational cost increases proportionally to the total number of *occurrences *of reaction or diffusion events. In order to capture the long time behavior of a system with both 'fast' and 'slow' reactions, such as a signal transduction network coupled with gene expression, these methods will spend the majority of their computational time simulating the occurrences of the fast reactions in the signalling network while only rarely executing a reaction event related to gene expression. The costs only increase when diffusion events are included. Because most biological systems feature widely disparate timescales, it becomes impractical to use the exact method to simulate a large, realistic biological system with many reactions and chemical species. This obstacle has motivated the development and use of approximate and hybrid stochastic methods.

Hy3S (pronounced hi-three-ess), or Hybrid Stochastic Simulation for Supercomputers, is an open sourced software package written for the development, dissemination, and productive use of hybrid stochastic simulation methods. The goal of the software is allow users to utilize the most recently developed stochastic methods to simulate extremely large, realistic biological systems. The software package includes multiple different hybrid stochastic simulation methods and a simple Matlab (Mathworks) driven GUI. It uses the NetCDF (Unidata) [16] interface to store both model and solution data in an optimized, platform-independent, array-based, binary format. By combining Matlab's built-in scripting language and data analysis functions, NetCDF's ability to quickly read or write *terabytes *of data, and our recently developed MPI-parallelized hybrid stochastic simulation method, it is now straightforward to simulate the stochastic dynamics of any extremely large, *arbitrary *biochemical reaction network with widely disparate time scales. The targeted production platform is an MPI-enabled Intel Itanium2 computing cluster running Linux, but the simulation programs have also run on x86, IBM, Cray, and SGI Altix computing platforms. The average user will most likely be a scientist, engineer, or mathematician who is already familiar with computational modelling.

Many approximate or hybrid stochastic methods that decrease the computational cost of stochastic simulation have been proposed [17,18]. Here, we focus on hybrid stochastic methods. A good hybrid stochastic method for simulating chemical kinetics partitions a system of reactions into multiple subsets, describes the time evolution of each subset as a different valid mathematical representation, and self-consistently merges all of the representations in order to produce an accurate solution and minimize the computational costs. Because the subsets of reactions are usually coupled, the challenge is to simultaneously solve different types of mathematical processes, including the coupling effects. In recent years, a few hybrid stochastic methods have been proposed [19-21]. While these methods each advance the state of the art, the goal of creating a replacement for the original stochastic simulation algorithm that quickly and accurately simulates large *arbitrary *reaction networks, possibly featuring dynamical stiffness or widely disparate timescales, has not been achieved. In general, many software packages have been developed to simulate the dynamics of biochemical networks, using both deterministic and stochastic methods [22-25] (and all references therein).

In our recent work on developing hybrid stochastic methods, we have progressed much closer to this goal by efficiently and accurately simulating a coupled jump/continuous Markov process [26] with many *thousands *of reactions and unique chemical species. Importantly, the method uses the recently derived differential Jump equations, a type of stochastic differential equation (SDE), to compute the times at which the slow reactions occur. These Jump equations tie together a jump and continuous Markov process by computing both the fast and slow dynamics through the simultaneous solution of a system of SDEs. The established connection between the solution of a hybrid jump/continuous Markov process and the well developed theory of stochastic differential equations places the numerical method on solid ground, enables the usage of implicit, higher order, and adaptive numerical integration methods, and allows characterization of both the local and global error of the solution. Without such a connection, it is difficult to escape the usage of hand waving. We briefly describe the details of the numerical algorithm in the Methods section. Here, we will focus on the features of the newly developed software package and its capabilities.

## Implementation

### Overview of the software design

The main component of the software package is the collection of simulation programs written in Fortran95/2 k and parallelized using MPI. Each simulation program accepts a NetCDF input file containing all of the model data, simulates the stochastic dynamics of the model, and places the solution data back in the NetCDF file. The NetCDF file format is open, self-describing, and has APIs in numerous programming languages, allowing anyone to create the input model data or analyze the output solution data using a variety of different programs. To assist users in quickly creating biochemical networks, we include a simple Matlab-driven GUI to create NetCDF input files. Users may also use Matlab's scripting language to compose NetCDF files, allowing for the complex construction of large networks. For data analysis and plotting, one may read the solution data back into Matlab and use its capable functions. Importantly, while we use Matlab for creating biochemical networks and analyzing data, both the simulation programs and the NetCDF file format are completely open. This enables us to focus our research and development on creating the fastest and most accurate hybrid stochastic methods while using existing or future tools to create complex biochemical networks and analyze solution data.

### The simulation programs

The collection of simulation programs include four different numerical implementations of a hybrid jump/continuous Markov stochastic simulator [26], abbreviated as HyJCMSS, and also the Next Reaction variant of the original stochastic simulation algorithm [13]. Each simulation program is parallelized with MPI, creating a total of ten different simulation programs. Extensive accuracy and speed testing has demonstrated that HyJCMSS is currently the most efficient and accurate hybrid stochastic numerical method, especially when simulating extremely large reaction networks. We will briefly cover the different numerical implementations of the algorithm. We emphasize, however, that each program can be effectively treated as a 'black box' and used for productive research by those who are not interested in the details behind each numerical method.

#### Overview of numerical methods

The hybrid jump/continuous Markov stochastic simulator dynamically partitions a system of reactions into 'fast/continuous' and 'slow/discrete' subsets, describes the effects of the fast/continuous reactions using the chemical Langevin equation [27], and computes the times of the slow reactions using the zero crossings of a system of Jump equations [26]. The chemical Langevin equation and the differential Jump equations are both Itô type stochastic differential equations (SDEs) and are numerically integrated using stochastic numerical integrators. The approximation of fast/continuous reactions as a continuous Markov process greatly increases the efficiency of simulation when compared to an exact jump Markov simulation. The accuracy of the approximation is controlled by two parameters, *ε *and *λ*, which completely parameterize the continuity approximation for any reaction network.

In addition, by allowing multiple occurrences of slow reactions in between numerical integrations of the chemical Langevin equation, the efficiency of the simulation of large biochemical networks is dramatically increased. However, when using this 'Multiple Slow Reaction' (MSR) approximation [26] on systems with highly mixed timescales, the accuracy of the solution will be affected. We include an MSR tolerance that determines the maximum effect of the MSR approximation. The tolerance slides between zero and one, from turning off the approximation to the blind use of it. For efficiently and accurately simulating systems with possibly mixed timescales the default value of 1/*ε *provides an optimal trade off.

The numerical theory behind the integration of SDEs differs significantly from deterministic differential equations, especially for higher order, implicit, and adaptive integration methods. For an excellent reference, see Kloeden & Platen [28]. For our purposes, the system of SDEs is typically non-linear, multiplicative, non-commutative, and contains multiple Wiener processes, which are also known as Brownian paths. We compute the solution of the system of SDEs using four different numerical methods of the strong type. Specifically, we include a fixed time step Euler-Maruyama method, a fixed time step Milstein method, an adaptive time step Euler-Maruyama method, and an adaptive time step Milstein method. However, we only include the adaptive Euler-Maruyama method for educational purposes because it may converge to an incorrect solution [29] and demonstrates the danger of glossing over the differences between stochastic and deterministic numerical methods.

The Euler-Maruyama method includes only the drift and diffusion terms of an Itô-Taylor expansion around the solution, giving the method a local strong error of O(√Δt). The Milstein method includes the drift, diffusion, and also a third term that requires the strong approximation of multiple two-dimensional Wiener integrals, decreasing the local strong error to O(Δt). However, because the evaluation of these two dimensional Wiener integrals is computationally intensive, the efficiency of the simulation may be decreased.

While fixed time step methods for stochastic numerical integrators are better characterized, it is possible to use an adaptive time step method to increase the efficiency of simulation, especially when dealing with dynamically stiff systems. Adaptive time step methods dynamically determine an optimal time step for the numerical integrator and are preferred when solving a system of SDEs that feature transient or intermittent dynamical stiffness. An adaptive time step method will decrease the time step of the numerical integration when dynamical stiffness exists, but will increase it when the system is no longer stiff. Adaptive time step methods for computing strong solutions of SDEs differ significantly from their deterministic counterparts. For example, when computing an optimal time step, the paths of the Brownian process must be conditioned on previous and future realized points of the paths. Otherwise, the solution will use a new Brownian path whose increments will be biased by the criteria for time step selection, resulting in a loss of accuracy. The criteria for optimal time step selection require a measure of the local error of the solution. We use previously proposed criteria [30] that measure the local error in both the drift and diffusion terms. We also apply an additional criterion that requires the fast/continuous reactions to remain validly approximated as a continuous Markov process during the time interval of numerical integration. We use the evaluation of the criteria to either decrease or increase the time step, as needed. A more detailed description of adaptive time step methods for SDEs is also available [29,30]. An overview of the advantages and disadvantages of each numerical method is shown in Table 1. We briefly describe both the fixed and adaptive forms of the Euler-Maruyama and Milstein methods below.

**Table 1:** An overview of the Hy3S numerical methods

Name	Advantages	Disadvantages
Next Reaction variant of SSA	Essentially exact	Extremely slow for 'large' systems
HyJCMSS Fixed Euler-Maruyama	HyJCMSS methods are much faster for 'large' systems. Fastest SDE numerical integrator for non-stiff systems.	For stiff systems, species populations may go negative. Finding an accurate time step for a system can be annoying.
HyJCMSS Fixed Milstein	Increased accuracy. May use a larger time step.	Evaluation of 2D Itô integrals decreases speed of simulation.
HyJCMSS Adaptive Euler-Maruyama	Automatically chooses an accurate time step, based on the SDE tolerance.	Does not always converge to the correct solution. Usage is inadvisable. Included for educational purposes only.
HyJCMSS Adaptive Milstein	Dynamically chooses accurate time step. Increased efficiency when transient stiffness exists. With a reasonable tolerance, convergence to correct solution is guaranteed.	Slower than fixed methods for systems with constant timescales, due to the computational overhead in the adaptive code.

Each simulation program has a number of command-line parameters that enable the user to tailor the accuracy and efficiency of the simulation for a particular system. However, the user may elect to use default values of the parameters and obtain a reasonably accurate and efficiently computed solution. A description of the parameters is shown in Table 2.

**Table 2:** A description of each command line argument and their default values

Command line parameter	Description	Which methods use it	Default value
Filename	NetCDF file name	All	None
Random Seed	The random number generator's seed value.	All	Randomized (system dependent)
Epsilon (*ε*)	Minimum number of molecules of both reactant & product species for approximation to a continuous Markov process	All HyJCMSS simulation methods	100 molecules
Lambda (*λ*)	Minimum rate of reaction for approximation to a continuous Markov process	All HyJCMSS simulation methods	10 molecules/sec
MSR Tolerance	Maximum relative effect of slow reactions per numerical integration of SDEs	All HyJCMSS simulation methods	1/*ε*
SDE Tolerance	Maximum values of drift& diffusion error criteria	Adaptive Methods	1e-4
SDE time step	Maximum time step of numerical integrator	Fixed Methods	0.10 seconds or Save Time

#### Optimizing data structures

In the simulation programs, there are three types of numerical operations where an optimized data structure increases computational performance. When solving the system of SDEs, we take advantage of the sparseness of both the stoichiometric matrix and the system of two dimensional stochastic integrals. By creating indexes and inverse indexes that map the full system to a reduced one, we minimize the computation of the solution of the SDEs. We also use a dependency graph to only compute reaction propensities and the derivatives of reaction propensities when they may have changed, similar to the dependency graph of the Next Reaction variant of the stochastic simulation algorithm [13], but extended to include the presence of fast/continuous reactions and special events, which are described below. Finally, we use an indexed priority queue to determine the reaction time corresponding to the next zero crossing of the differential Jump equations and a sorted queue to determine the next special event time that may occur. These optimizing data structures increase the efficiency of simulation, especially for systems with numerous reactions and chemical species.

#### MPI parallelization

Each simulation program is embarrassingly parallelized using MPI. Typically, numerous independent realizations of the stochastic dynamics of a biochemical network are desired. Each processor is allocated a number of independent trials to simulate. If the number of independent trials is divisible by the number of processors then the efficiency of the implementation is near 100%. Parallel write and read access is utilized so that no processor writes to the same portion of the NetCDF at any time and all processors may read the NetCDF at any time. For example, if 10 000 independent stochastic simulations of a biochemical network are desired and 500 processors are allocated to the simulation program then the computational time of the program will, in fact, be reduced by about 500 times. Computing clusters with thousands of processors, such as the NSF supported TeraGrid [31], enable this high level of research productivity.

Because we use MPI as our underlying programming model for processor-to-processor communication, we can also readily extend each simulation program's usage to a grid computing environment, such as one utilizing MPICH-G2 [32]. A grid computing environment allows a program to utilize computing processors from multiple different computers. The computers may be geographically separated and joined with a high bandwidth, but relatively high latency, communications connection. Due to the high latency, it is important to minimize the blocking processor-to-processor communication, which is achieved with our current implementation. Running a program on a previously configured grid environment can be as simple as constructing a short script and executing a command.

#### Solution of a hybrid jump/continuous Markov process

Consider a homogeneous system of M reactions and N species with a stoichiometric matrix, v, a vector of reaction propensities, a, and a state vector, X, consisting of the number of molecules of each species. The system is dynamically partitioned into fast/continuous and slow reaction subsets with M^fast ^and M^slow ^reactions, respectively. The j^th ^reaction is classified as fast/continuous if and only if the following is true:

*a_j _*(*t*) ≥ >> 1

*X_i _*(*t*) > *ε*·|*v_ji_*| *i *∈ {reactant or product of j^th ^reaction}'     (1)

where *ε *and *λ *are the parameters described in the above sections. The above criterion is evaluated with each iteration of the algorithm. The reaction propensities for the fast/continuous and slow reactions are respectively labeled a^f ^and a^s^.

The fast/continuous reactions are approximated as a continuous Markov process by performing an Ω-expansion of the governing Master equation and deriving a multi-dimensional Fokker-Planck equation [39]. The Fokker-Planck equation describes the evolution of the probability distribution of the system considering the effects of only the fast/continuous reactions. Or, equivalently, one can determine a system of Itô stochastic differential equations that describes how the state of the system evolves over time, ignoring the occurrences of the slow reactions. For chemical reaction networks, the system of Itô stochastic differential equations is named the chemical Langevin equation [27], stated as

dXi=∑j=1Mfastvjiajf(X¯(t))dt+∑j=1Mfastvjiajf(X¯(t))dWj,     (2)
 MathType@MTEF@5@5@+=feaafiart1ev1aaatCvAUfKttLearuWrP9MDH5MBPbIqV92AaeXatLxBI9gBaebbnrfifHhDYfgasaacH8akY=wiFfYdH8Gipec8Eeeu0xXdbba9frFj0=OqFfea0dXdd9vqai=hGuQ8kuc9pgc9s8qqaq=dirpe0xb9q8qiLsFr0=vr0=vr0dc8meaabaqaciaacaGaaeqabaqabeGadaaakeaacqWGKbazcqWGybawdaWgaaWcbaGaemyAaKgabeaakiabg2da9maaqahabaGaemODay3aaSbaaSqaaiabdQgaQjabdMgaPbqabaGccqWGHbqydaqhaaWcbaGaemOAaOgabaGaemOzaygaaOGaeiikaGIafmiwaGLba0bacqGGOaakcqWG0baDcqGGPaqkcqGGPaqkcqWGKbazcqWG0baDcqGHRaWkdaaeWbqaaiabdAha2naaBaaaleaacqWGQbGAcqWGPbqAaeqaaOWaaOaaaeaacqWGHbqydaqhaaWcbaGaemOAaOgabaGaemOzaygaaOGaeiikaGIafmiwaGLba0bacqGGOaakcqWG0baDcqGGPaqkcqGGPaqkaSqabaaabaGaemOAaOMaeyypa0JaeGymaedabaGaemyta00aaWbaaWqabeaacqWGMbGzcqWGHbqycqWGZbWCcqWG0baDaaaaniabggHiLdaaleaacqWGQbGAcqGH9aqpcqaIXaqmaeaacqWGnbqtdaahaaadbeqaaiabdAgaMjabdggaHjabdohaZjabdsha0baaa0GaeyyeIuoakiabdsgaKjabdEfaxnaaBaaaleaacqWGQbGAaeqaaOGaeiilaWIaaCzcaiaaxMaacqGGOaakcqaIYaGmcqGGPaqkaaa@74CC@

where the dW refers to a vector of Wiener increments of the multidimensional Wiener process.

The slow reactions are described as a jump Markov process, whose events have waiting times that are distributed according to a time-dependent probability distribution. The time dependence arises from changes in the state vector due to the numerical integration of the chemical Langevin equation. In order to explicitly account for this time-dependence, we have previously derived [26] a system of differential Jump equations,

dRjdt=ajs(t),Rj(to)=log⁡(URNj),j=1...Mslow,     (3)
 MathType@MTEF@5@5@+=feaafiart1ev1aaatCvAUfKttLearuWrP9MDH5MBPbIqV92AaeXatLxBI9gBaebbnrfifHhDYfgasaacH8akY=wiFfYdH8Gipec8Eeeu0xXdbba9frFj0=OqFfea0dXdd9vqai=hGuQ8kuc9pgc9s8qqaq=dirpe0xb9q8qiLsFr0=vr0=vr0dc8meaabaqaciaacaGaaeqabaqabeGadaaakeaafaqabeqadaaabaWaaSaaaeaacqWGKbazcqWGsbGudaWgaaWcbaGaemOAaOgabeaaaOqaaiabdsgaKjabdsha0baacqGH9aqpcqWGHbqydaqhaaWcbaGaemOAaOgabaGaem4CamhaaOGaeiikaGIaemiDaqNaeiykaKIaeiilaWcabaGaemOuai1aaSbaaSqaaiabdQgaQbqabaGccqGGOaakcqWG0baDdaWgaaWcbaGaem4Ba8gabeaakiabcMcaPiabg2da9iGbcYgaSjabc+gaVjabcEgaNjabcIcaOiabdwfavjabdkfasjabd6eaonaaBaaaleaacqWGQbGAaeqaaOGaeiykaKIaeiilaWcabaGaemOAaOMaeyypa0JaeGymaeJaeiOla4IaeiOla4IaeiOla4Iaemyta00aaWbaaSqabeaacqWGZbWCcqWGSbaBcqWGVbWBcqWG3bWDaaGccqGGSaalaaGaaCzcaiaaxMaadaqadaqaaiabiodaZaGaayjkaiaawMcaaaaa@62EB@

which describe the rate of change of a system of reaction residuals, R. A slow reaction occurs when its corresponding reaction residual performs a zero crossings from a negative to positive value. By numerically integrating the system of differential Jump equations along with the chemical Langevin equation and monitoring the zero crossings of the reaction residuals, one can account for the coupled nature of the jump and continuous Markov processes and accurately compute the times of the slow reactions. One can also perform an Itô-Taylor expansion around each reaction residual to predict the time of the next zero crossing. Note that, because of their dependence on the state vector, the system of differential Jump equations are also Itô stochastic differential equations, but do not contain a Wiener process. In addition, if the jump and continuous Markov processes are uncoupled or if there are only slow reactions, then the system of differential Jump equations simplify to the Next Reaction variant [13] method of computing the reaction times.

By using stochastic differential equations to describe both the *effects *of the fast/continuous reactions and the *times *of the slow reactions, we now have a considerable amount of numerical integration theory at our disposal. We have implemented four different stochastic numerical integrators to compute the solution of Eqs (2) and (3), both fixed and adaptive schemes. When we use a fixed scheme to numerically integrate Eqs (2) and (3) we must include the Multiple Slow Reaction (MSR) approximation criteria in a special fashion. An efficient method of including the tolerance is to numerically integrate Eq. (3) forward in time until either the number of zero crossings has maximally satisfied the MSR tolerance value or the time step is equal to the chosen fixed time step. One then uses the same time step to numerically integrate Eq. (2). If the MSR tolerance is small, the time step may change during the simulation. However, the method still has the properties of a fixed scheme because the Wiener increments are only evaluated once and no conditional probabilities are required. The global error of this scheme is no worse than a normal fixed scheme because enforcement of the MSR tolerance always decreases the utilized time step, generating a smaller local error. When using an adaptive scheme to solve Eqs. (2) and (3), we simply half or double the time step based on the MSR criteria in addition to the other criteria.

We now briefly describe each numerical integration scheme we use, but note that more comprehensive derivations of these methods are available[28].

#### The fixed euler-maruyama method

The Euler-Maruyama method is an explicit stochastic numerical integration method with strong accuracy of order O(√Δt). It is derived from the Itô-Taylor expansion around the solution, truncating after the diffusion term. Applied to the chemical Langevin equation, it is stated as

Xik+1=Xik+∑j=1Mfastvjiajf(X¯k)Δt+∑j=1Mfastvjiajf(X¯k)ΔWj(1),     (4)
 MathType@MTEF@5@5@+=feaafiart1ev1aaatCvAUfKttLearuWrP9MDH5MBPbIqV92AaeXatLxBI9gBaebbnrfifHhDYfgasaacH8akY=wiFfYdH8Gipec8Eeeu0xXdbba9frFj0=OqFfea0dXdd9vqai=hGuQ8kuc9pgc9s8qqaq=dirpe0xb9q8qiLsFr0=vr0=vr0dc8meaabaqaciaacaGaaeqabaqabeGadaaakeaacqWGybawdaqhaaWcbaGaemyAaKgabaGaem4AaSMaey4kaSIaeGymaedaaOGaeyypa0JaemiwaG1aa0baaSqaaiabdMgaPbqaaiabdUgaRbaakiabgUcaRmaaqahabaGaemODay3aaSbaaSqaaiabdQgaQjabdMgaPbqabaGccqWGHbqydaqhaaWcbaGaemOAaOgabaGaemOzaygaaOGaeiikaGIafmiwaGLba0badaahaaWcbeqaaiabdUgaRbaakiabcMcaPiabgs5aejabdsha0jabgUcaRaWcbaGaemOAaOMaeyypa0JaeGymaedabaGaemyta00aaWbaaWqabeaacqWGMbGzcqWGHbqycqWGZbWCcqWG0baDaaaaniabggHiLdGcdaaeWbqaaiabdAha2naaBaaaleaacqWGQbGAcqWGPbqAaeqaaOWaaOaaaeaacqWGHbqydaqhaaWcbaGaemOAaOgabaGaemOzaygaaOGaeiikaGIafmiwaGLba0badaahaaWcbeqaaiabdUgaRbaakiabcMcaPaWcbeaaaeaacqWGQbGAcqGH9aqpcqaIXaqmaeaacqWGnbqtdaahaaadbeqaaiabdAgaMjabdggaHjabdohaZjabdsha0baaa0GaeyyeIuoakiabgs5aeHqaaiab=DfaxnaaDaaaleaacqWFQbGAaeaacqWFOaakcqWFXaqmcqWFPaqkaaGccqGGSaalcaWLjaGaaCzcaiabcIcaOiabisda0iabcMcaPaaa@7B6E@

where ΔW^(1)^_j _is a normal Gaussian random number with a mean of zero and a variance of Δt. Applied to the differential Jump equations, the scheme is simply

Rjk+1=Rjk+ajs(X¯k)Δt.     (5)
 MathType@MTEF@5@5@+=feaafiart1ev1aaatCvAUfKttLearuWrP9MDH5MBPbIqV92AaeXatLxBI9gBaebbnrfifHhDYfgasaacH8akY=wiFfYdH8Gipec8Eeeu0xXdbba9frFj0=OqFfea0dXdd9vqai=hGuQ8kuc9pgc9s8qqaq=dirpe0xb9q8qiLsFr0=vr0=vr0dc8meaabaqaciaacaGaaeqabaqabeGadaaakeaacqWGsbGudaqhaaWcbaGaemOAaOgabaGaem4AaSMaey4kaSIaeGymaedaaOGaeyypa0JaemOuai1aa0baaSqaaiabdQgaQbqaaiabdUgaRbaakiabgUcaRiabdggaHnaaDaaaleaacqWGQbGAaeaacqWGZbWCaaGccqGGOaakcuWGybawgaqhamaaCaaaleqabaGaem4AaSgaaOGaeiykaKIaeyiLdqKaemiDaqNaeiOla4IaaCzcaiaaxMaacqGGOaakcqaI1aqncqGGPaqkaaa@4943@

#### The fixed milstein method

The Milstein method is an explicit stochastic numerical integrator with strong accuracy of O(Δt). The increased accuracy originates from retaining terms of O(Δt) in the Itô-Taylor expansion around the solution. These additional terms contain two-dimensional stochastic integrals, which are defined as

I(j1,j2)=∫tt+ΔtdW(t)j1dW(t)j2.     (6)
MathType@MTEF@5@5@+=feaafiart1ev1aaatCvAUfKttLearuWrP9MDH5MBPbIqV92AaeXatLxBI9gBaebbnrfifHhDYfgasaacH8akY=wiFfYdH8Gipec8Eeeu0xXdbba9frFj0=OqFfea0dXdd9vqai=hGuQ8kuc9pgc9s8qqaq=dirpe0xb9q8qiLsFr0=vr0=vr0dc8meaabaqaciaacaGaaeqabaqabeGadaaakeaacqWGjbqscqGGOaakcqWGQbGAdaWgaaWcbaGaeGymaedabeaakiabcYcaSiabdQgaQnaaBaaaleaacqaIYaGmaeqaaOGaeiykaKIaeyypa0Zaa8qCaeaacqWGKbazcqWGxbWvcqGGOaakcqWG0baDcqGGPaqkdaWgaaWcbaGaemOAaOMaeGymaedabeaakiabdsgaKjabdEfaxjabcIcaOiabdsha0jabcMcaPmaaBaaaleaacqWGQbGAcqaIYaGmaeqaaOGaeiOla4IaaCzcaiaaxMaacqGGOaakcqaI2aGncqGGPaqkaSqaaiabdsha0bqaaiabdsha0jabgUcaRiabgs5aejabdsha0bqdcqGHRiI8aaaa@549E@

The two dimensional stochastic integrals are used to describe the time evolution of a random variable that is dependent on multiple Wiener processes. For a single Wiener process or when j_1 _= j_2_, a single realization of Eq (6) simplifies to the evaluation of

I(j1,j1)=12{(ΔWj1)2−Δt}.     (7)
 MathType@MTEF@5@5@+=feaafiart1ev1aaatCvAUfKttLearuWrP9MDH5MBPbIqV92AaeXatLxBI9gBaebbnrfifHhDYfgasaacH8akY=wiFfYdH8Gipec8Eeeu0xXdbba9frFj0=OqFfea0dXdd9vqai=hGuQ8kuc9pgc9s8qqaq=dirpe0xb9q8qiLsFr0=vr0=vr0dc8meaabaqaciaacaGaaeqabaqabeGadaaakeaacqWGjbqscqGGOaakcqWGQbGAdaWgaaWcbaGaeGymaedabeaakiabcYcaSiabdQgaQnaaBaaaleaacqaIXaqmaeqaaOGaeiykaKIaeyypa0ZaaSaaaeaacqaIXaqmaeaacqaIYaGmaaWaaiWaaeaadaqadaqaaiabgs5aejabdEfaxnaaBaaaleaacqWGQbGAcqaIXaqmaeqaaaGccaGLOaGaayzkaaWaaWbaaSqabeaacqaIYaGmaaGccqGHsislcqGHuoarcqWG0baDaiaawUhacaGL9baacqGGUaGlcaWLjaGaaCzcaiabcIcaOiabiEda3iabcMcaPaaa@4AF6@

However, for multiple different Wiener processes, there is no analytic expression for realizations of Eq (6). Instead, once can Fourier expand Eq. (6) in terms of Gaussian distributed coefficients and generate strong approximations, using

I(j1,j2)=Δt{12ξj1ξj2+ρ P(μj1ξj2−μj2ξj1)}+Δt2π∑r=1P1r{ζj1,r(2ξj2+ηj2,r)−ζj2,r(2ξj1+ηj1,r)}     (8)
 MathType@MTEF@5@5@+=feaafiart1ev1aaatCvAUfKttLearuWrP9MDH5MBPbIqV92AaeXatLxBI9gBaebbnrfifHhDYfgasaacH8akY=wiFfYdH8Gipec8Eeeu0xXdbba9frFj0=OqFfea0dXdd9vqai=hGuQ8kuc9pgc9s8qqaq=dirpe0xb9q8qiLsFr0=vr0=vr0dc8meaabaqaciaacaGaaeqabaqabeGadaaakqaabeqaaiabdMeajjabcIcaOiabdQgaQnaaBaaaleaacqaIXaqmaeqaaOGaeiilaWIaemOAaO2aaSbaaSqaaiabikdaYaqabaGccqGGPaqkcqGH9aqpcqGHuoarcqWG0baDdaGadaqaamaalaaabaGaeGymaedabaGaeGOmaidaaGGaciab=57a4naaBaaaleaacqWGQbGAcqaIXaqmaeqaaOGae8NVdG3aaSbaaSqaaiabdQgaQjabikdaYaqabaGccqGHRaWkdaGcaaqaaiab=f8aYnaaBaaaleaacaaMc8UaemiuaafabeaaaeqaaOWaaeWaaeaacWaJa+hVd02aiWiGBaaaleacmcOamWiGdQgaQjadmciIXaqmaeqcmciakiadmc4F+oaEdGaJaUbaaSqaiWiGcWaJaoOAaOMamWiGikdaYaqajWiGaOGaeyOeI0Iae8hVd02aaSbaaSqaaiabdQgaQjabikdaYaqabaGccqWF+oaEdaWgaaWcbaGaemOAaOMaeGymaedabeaaaOGaayjkaiaawMcaaaGaay5Eaiaaw2haaaqaaiabgUcaRmaalaaabaGaeyiLdqKaemiDaqhabaGaeGOmaiJae8hWdahaamaaqahabaWaaSaaaeaacqaIXaqmaeaacqWGYbGCaaWaaiWaaeaacqWF2oGEdaWgaaWcbaGaemOAaOMaeGymaeJaeiilaWIaemOCaihabeaakmaabmaabaWaaOaaaeaacqaIYaGmaSqabaGccqWF+oaEdaWgaaWcbaGaemOAaOMaeGOmaidabeaakiabgUcaRiab=D7aOnaaBaaaleaacqWGQbGAcqaIYaGmcqGGSaalcqWGYbGCaeqaaaGccaGLOaGaayzkaaGaeyOeI0Iae8NTdO3aaSbaaSqaaiabdQgaQjabikdaYiabcYcaSiabdkhaYbqabaGcdaqadaqaamaakaaabaGaeGOmaidaleqaaOGae8NVdG3aaSbaaSqaaiabdQgaQjabigdaXaqabaGccqGHRaWkcqWF3oaAdaWgaaWcbaGaemOAaOMaeGymaeJaeiilaWIaemOCaihabeaaaOGaayjkaiaawMcaaaGaay5Eaiaaw2haaaWcbaGaemOCaiNaeyypa0JaeGymaedabaGaemiuaafaniabggHiLdGccaWLjaGaaCzcaiabcIcaOiabiIda4iabcMcaPaaaaa@AC39@

with a P-dependent constant ρ P=112−12π2∑r=1P1r2,
 MathType@MTEF@5@5@+=feaafiart1ev1aaatCvAUfKttLearuWrP9MDH5MBPbIqV92AaeXatLxBI9gBaebbnrfifHhDYfgasaacH8akY=wiFfYdH8Gipec8Eeeu0xXdbba9frFj0=OqFfea0dXdd9vqai=hGuQ8kuc9pgc9s8qqaq=dirpe0xb9q8qiLsFr0=vr0=vr0dc8meaabaqaciaacaGaaeqabaqabeGadaaakeaaiiGacqWFbpGCdaWgaaWcbaGaaGPaVlabdcfaqbqabaGccqGH9aqpdaWcaaqaaiabigdaXaqaaiabigdaXiabikdaYaaacqGHsisldaWcaaqaaiabigdaXaqaaiabikdaYiab=b8aWnaaCaaaleqabaGaeGOmaidaaaaakmaaqahabaWaaSaaaeaacqaIXaqmaeaacqWGYbGCdaahaaWcbeqaaiabikdaYaaaaaGccqGGSaalaSqaaiabdkhaYjabg2da9iabigdaXaqaaiabdcfaqbqdcqGHris5aaaa@4649@

where *ζ*_*j*,*r*_, *η*_*j*,*r *_and *μ*_*j *_are independent normal Gaussian random numbers, N(0,1), and *ξ*_*j *_is related to the Wiener increments via ξj=ΔWjΔt
 MathType@MTEF@5@5@+=feaafiart1ev1aaatCvAUfKttLearuWrP9MDH5MBPbIqV92AaeXatLxBI9gBaebbnrfifHhDYfgasaacH8akY=wiFfYdH8Gipec8Eeeu0xXdbba9frFj0=OqFfea0dXdd9vqai=hGuQ8kuc9pgc9s8qqaq=dirpe0xb9q8qiLsFr0=vr0=vr0dc8meaabaqaciaacaGaaeqabaqabeGadaaakeaaiiGacqWF+oaEdaWgaaWcbaGaemOAaOgabeaakiabg2da9maalaaabaGaeyiLdqKaem4vaC1aaSbaaSqaaiabdQgaQbqabaaakeaadaGcaaqaaiabgs5aejabdsha0bWcbeaaaaaaaa@3843@, for j = 1 ... M^fast ^and r = 1 ... P. The constant P arises from the number of retained terms in the Fourier expansion and controls the accuracy of the approximation. We set P to 10 to generate reasonably accurate realizations of the two dimensional stochastic integrals without requiring an excessive number of normal Gaussian random numbers.

Applied to the chemical Langevin equations, the Milstein scheme is stated as

Xik+1=Xik+∑j=1Mfasstvjiajf(X¯k)Δt+∑j=1Mfastvjiajf(X¯k)ΔWj+12∑j1,j2=1Mfast∑n=1Nvj1nvj2iaj1(X¯k)aj2(X¯k)∂aj1∂XnI(j1,j2),     (9)
 MathType@MTEF@5@5@+=feaafiart1ev1aaatCvAUfKttLearuWrP9MDH5MBPbIqV92AaeXatLxBI9gBaebbnrfifHhDYfgasaacH8akY=wiFfYdH8Gipec8Eeeu0xXdbba9frFj0=OqFfea0dXdd9vqai=hGuQ8kuc9pgc9s8qqaq=dirpe0xb9q8qiLsFr0=vr0=vr0dc8meaabaqaciaacaGaaeqabaqabeGadaaakeaafaqadeGabaaabaGaemiwaG1aa0baaSqaaiabdMgaPbqaaiabdUgaRjabgUcaRiabigdaXaaakiabg2da9iabdIfaynaaDaaaleaacqWGPbqAaeaacqWGRbWAaaGccqGHRaWkdaaeWbqaaiabdAha2naaBaaaleaacqWGQbGAcqWGPbqAaeqaaOGaemyyae2aa0baaSqaaiabdQgaQbqaaiabdAgaMbaakiabcIcaOiqbdIfayzaaDaWaaWbaaSqabeaacqWGRbWAaaGccqGGPaqkcqGHuoarcqWG0baDaSqaaiabdQgaQjabg2da9iabigdaXaqaaiabd2eannaaCaaameqabaGaemOzayMaemyyaeMaem4CamNaem4CamNaemiDaqhaaaqdcqGHris5aOGaey4kaSYaaabCaeaacqWG2bGDdaWgaaWcbaGaemOAaOMaemyAaKgabeaakmaakaaabaGaemyyae2aa0baaSqaaiabdQgaQbqaaiabdAgaMbaakiabcIcaOiqbdIfayzaaDaWaaWbaaSqabeaacqWGRbWAaaGccqGGPaqkaSqabaGccqGHuoarcqWGxbWvdaWgaaWcbaGaemOAaOgabeaaaeaacqWGQbGAcqGH9aqpcqaIXaqmaeaacqWGnbqtdaahaaadbeqaaiabdAgaMjabdggaHjabdohaZjabdsha0baaa0GaeyyeIuoaaOqaaiabgUcaRmaalaaabaGaeGymaedabaGaeGOmaidaamaaqahabaWaaabCaeaacqWG2bGDdaWgaaWcbaGaemOAaO2aaSbaaWqaaiabigdaXaqabaWccqWGUbGBaeqaaOGaemODay3aaSbaaSqaaiabdQgaQnaaBaaameaacqaIYaGmaeqaaSGaemyAaKgabeaakmaakaaabaWaaSaaaeaacqWGHbqydaWgaaWcbaGaemOAaO2aaSbaaWqaaiabigdaXaqabaaaleqaaOGaeiikaGIafmiwaGLba0badaahaaWcbeqaaiabdUgaRbaakiabcMcaPaqaaiabdggaHnaaBaaaleaacqWGQbGAdaWgaaadbaGaeGOmaidabeaaaSqabaGccqGGOaakcuWGybawgaqhamaaCaaaleqabaGaem4AaSgaaOGaeiykaKcaaaWcbeaakmaalaaabaacciGae8NaIyRaemyyae2aaSbaaSqaaiabdQgaQnaaBaaameaacqaIXaqmaeqaaaWcbeaaaOqaaiab=jGi2kabdIfaynaaBaaaleaacqWGUbGBaeqaaaaakiabdMeajjabcIcaOiabdQgaQnaaBaaaleaacqaIXaqmaeqaaOGaeiilaWIaemOAaO2aaSbaaSqaaiabikdaYaqabaGccqGGPaqkaSqaaiabd6gaUjabg2da9iabigdaXaqaaiabd6eaobqdcqGHris5aaWcbaGaemOAaO2aaSbaaWqaaiabigdaXaqabaWccqGGSaalcqWGQbGAdaWgaaadbaGaeGOmaidabeaaliabg2da9iabigdaXaqaaiabd2eannaaCaaameqabaGaemOzayMaemyyaeMaem4CamNaemiDaqhaaaqdcqGHris5aaaakiabcYcaSiaaxMaacaWLjaWaaeWaaeaacqaI5aqoaiaawIcacaGLPaaaaaa@C3E5@

where the first summation of the third term is taken over all possible combinations of a pair of fast/continuous reactions. Conveniently, because the stoichiometric matrix is typically sparse, there will be many zeros in the summations of the third term. After classifying reactions as fast/continuous or slow we create an index of all non-zero values and only compute the needed two dimensional stochastic integrals and coefficients. Because the differential Jump equations lack a Wiener process, the Milstein scheme applied to them is the same as in Eq. (5).

#### Adaptive methods

Our implementation of an adaptive time step scheme involves a three step process: evaluation of criteria that measure the local error of the solution, the halving or doubling of the time step according to the value of the criteria, and the determination of the Wiener increments corresponding to the decreased or increased time step. We restrict ourselves to only halving or doubling the time step for two reasons. The first is that the structure of the Brownian bridge becomes a Brownian binary tree and allows for easy storage and retrieval. The second is that, because the chemical Langevin equation contains multiple, non-commutative, multiplicative noise sources, it is not straightforward to calculate the necessary 2D stochastic integrals without simplifying the time step selection scheme.

We use a previously proposed set of criteria [30] that measure the local error in both the drift and diffusion components of the solution. The drift local error is measured by computing the difference between the Euler and Heun methods, which is

Ed(Xik,Δt)=‖Δt2(f′(X¯k+Δtf′(X¯k))−f′(X¯k))‖∞,     (10)
 MathType@MTEF@5@5@+=feaafiart1ev1aaatCvAUfKttLearuWrP9MDH5MBPbIqV92AaeXatLxBI9gBaebbnrfifHhDYfgasaacH8akY=wiFfYdH8Gipec8Eeeu0xXdbba9frFj0=OqFfea0dXdd9vqai=hGuQ8kuc9pgc9s8qqaq=dirpe0xb9q8qiLsFr0=vr0=vr0dc8meaabaqaciaacaGaaeqabaqabeGadaaakeaacqWGfbqrdaWgaaWcbaGaemizaqgabeaakiabcIcaOiabdIfaynaaDaaaleaacqWGPbqAaeaacqWGRbWAaaGccqGGSaalcqGHuoarcqWG0baDcqGGPaqkcqGH9aqpdaqbdaqaamaalaaabaGaeyiLdqKaemiDaqhabaGaeGOmaidaamaabmaabaGafmOzayMbauaadaqadaqaaiqdmc4GybawgGaJa2badGaJaYbaaSqajWiGbGaJakadmc4GRbWAaaGccqGHRaWkcqGHuoarcqWG0baDcuWGMbGzgaqbamaabmaabaGafmiwaGLba0badaahaaWcbeqaaiabdUgaRbaaaOGaayjkaiaawMcaaaGaayjkaiaawMcaaiabgkHiTiqbdAgaMzaafaWaaeWaaeaacuWGybawgaqhamaaCaaaleqabaGaem4AaSgaaaGccaGLOaGaayzkaaaacaGLOaGaayzkaaaacaGLjWUaayPcSdWaaSbaaSqaaiabg6HiLcqabaGccqGGSaalcaWLjaGaaCzcaiabcIcaOiabigdaXiabicdaWiabcMcaPaaa@667B@

where f′(x¯)
 MathType@MTEF@5@5@+=feaafiart1ev1aaatCvAUfKttLearuWrP9MDH5MBPbIqV92AaeXatLxBI9gBaebbnrfifHhDYfgasaacH8akY=wiFfYdH8Gipec8Eeeu0xXdbba9frFj0=OqFfea0dXdd9vqai=hGuQ8kuc9pgc9s8qqaq=dirpe0xb9q8qiLsFr0=vr0=vr0dc8meaabaqaciaacaGaaeqabaqabeGadaaakeaacuWGMbGzgaqbaiabcIcaOGqaaiqb=Hha4zaaDaGaeiykaKcaaa@3161@ is the matrix, ∂ajXi| x¯
 MathType@MTEF@5@5@+=feaafiart1ev1aaatCvAUfKttLearuWrP9MDH5MBPbIqV92AaeXatLxBI9gBaebbnrfifHhDYfgasaacH8akY=wiFfYdH8Gipec8Eeeu0xXdbba9frFj0=OqFfea0dXdd9vqai=hGuQ8kuc9pgc9s8qqaq=dirpe0xb9q8qiLsFr0=vr0=vr0dc8meaabaqaciaacaGaaeqabaqabeGadaaakeaadaWcaaqaaGGaciab=jGi2kabdggaHnaaBaaaleaacqWGQbGAaeqaaaGcbaGaemiwaG1aaSbaaSqaaiabdMgaPbqabaaaaOGaeiiFaW3aaSbaaSqaaiaaykW7cuWG4baEgaqhaaqabaaaaa@38A3@. The drift local error is of order O(Δt^2^). The diffusion local error is measured by performing an Itô-Taylor expansion of the Milstein scheme and selecting an O(Δt^3/2^) term that is most efficient to compute. The diffusion criteria is

E(Xik,Δt)=112‖(ΔWj3aj(X¯k))•(vji∂aj∂Xi|X¯k)‖∞‖vji∂aj∂Xi|X¯k‖∞.     (11)
 MathType@MTEF@5@5@+=feaafiart1ev1aaatCvAUfKttLearuWrP9MDH5MBPbIqV92AaeXatLxBI9gBaebbnrfifHhDYfgasaacH8akY=wiFfYdH8Gipec8Eeeu0xXdbba9frFj0=OqFfea0dXdd9vqai=hGuQ8kuc9pgc9s8qqaq=dirpe0xb9q8qiLsFr0=vr0=vr0dc8meaabaqaciaacaGaaeqabaqabeGadaaakeaacqWGfbqrcqGGOaakcqWGybawdaqhaaWcbaGaemyAaKgabaGaem4AaSgaaOGaeiilaWIaeyiLdqKaemiDaqNaeiykaKIaeyypa0ZaaSaaaeaacqaIXaqmaeaacqaIXaqmcqaIYaGmaaWaauWaaeaadaqadaqaamaalaaabaGaeyiLdqKaem4vaC1aa0baaSqaaiabdQgaQbqaaiabiodaZaaaaOqaamaakaaabaGaemyyae2aaSbaaSqaaiabdQgaQbqabaGccqGGOaakcuWGybawgaqhamaaCaaaleqabaGaem4AaSgaaOGaeiykaKcaleqaaaaaaOGaayjkaiaawMcaaiabgkci3oaabmaabaGaemODay3aaSbaaSqaaiabdMgaPjabdQgaQbqabaGcdaWcaaqaaGGaciab=jGi2kabdggaHnaaBaaaleaacqWGQbGAaeqaaaGcbaGae8NaIyRaemiwaG1aaSbaaSqaaiabdMgaPbqabaaaaOGaeiiFaW3aaSbaaSqaaiqbdIfayzaaDaWaaWbaaWqabeaacqWGRbWAaaaaleqaaaGccaGLOaGaayzkaaaacaGLjWUaayPcSdWaaSbaaSqaaiabg6HiLcqabaGcdaqbdaqaaiabdAha2naaBaaaleaacqWGPbqAcqWGQbGAaeqaaOWaaSaaaeaacqWFciITcqWGHbqydaWgaaWcbaGaemOAaOgabeaaaOqaaiab=jGi2kabdIfaynaaBaaaleaacqWGPbqAaeqaaaaakiabcYha8naaBaaaleaacuWGybawgaqhamaaCaaameqabaGaem4AaSgaaaWcbeaaaOGaayzcSlaawQa7amaaBaaaleaacqGHEisPaeqaaOGaeiOla4IaaCzcaiaaxMaacqGGOaakcqaIXaqmcqaIXaqmcqGGPaqkaaa@7F1A@

The max norm is taken to be the maximum absolute sum along the *j *dimension. A third criterion is that the numbers of molecules of all species affected by fast/continuous reactions must remain above a certain threshold, which is arbitrarily taken to be 20 molecules. The latter criterion will force the fast/continuous reactions to remain reasonably approximated as a continuous Markov process during the interval of numerical integration. We evaluate Eqs. (10) and (11) and also the third criterion for each species in the system and consider the local error to be small only when Eqs. (10) and (11) are less than the user-defined tolerance value and when the third criterion is true.

Time increments are described using a binary tree structure. The top node, or row, is the initial time step of the simulation. Additional nodes and rows are created by halving the time step of parent nodes. The number of nodes of each row is always 2^R-1 ^and the current time step is always Δt_o_/2^R-1^, where R is the row number, starting at one, and Δt_o _is the initial time step. If the local error is large, we halve the time step and move down a row. If the local error is small, we may double the time step and move up a row, but only if the number of the current node, or branch, is divisible by two. If the branch is not divisible by two, we simply maintain the time step. As we successfully numerically integrate forward in time, we increment the branch number.

The main reason for using the binary tree described above is to efficiently compute the Brownian bridges. At the top row of the tree, the Wiener increments for the initial time step are computed. When the time step is decreased we may not re-evaluate a Wiener increment for the halved time increment because the value of the Wiener process at the final time has already been evaluated. Instead, we must compute the Wiener increment of the halved time step, *conditioned *on the value of the beginning and ending Wiener process. To generate the intermediate Wiener increments, we use

ΔW2pr=12ΔWpr−1+γprp=0,1,2,3,...2r−2−1ΔW2p+1r=12ΔWpr−1−γprr=2,3,...,Rγpr=N(0,2−r),     (12)
 MathType@MTEF@5@5@+=feaafiart1ev1aaatCvAUfKttLearuWrP9MDH5MBPbIqV92AaeXatLxBI9gBaebbnrfifHhDYfgasaacH8akY=wiFfYdH8Gipec8Eeeu0xXdbba9frFj0=OqFfea0dXdd9vqai=hGuQ8kuc9pgc9s8qqaq=dirpe0xb9q8qiLsFr0=vr0=vr0dc8meaabaqaciaacaGaaeqabaqabeGadaaakeaafaqabeWacaaabaGaeyiLdqKaem4vaC1aa0baaSqaaiabikdaYiabdchaWbqaaiabdkhaYbaakiabg2da9maalaaabaGaeGymaedabaGaeGOmaidaaiabgs5aejabdEfaxnaaDaaaleaacqWGWbaCaeaacqWGYbGCcqGHsislcqaIXaqmaaGccqGHRaWkiiGacqWFZoWzdaqhaaWcbaGaemiCaahabaGaemOCaihaaaGcbaGaemiCaaNaeyypa0JaeGimaaJaeiilaWIaeGymaeJaeiilaWIaeGOmaiJaeiilaWIaeG4mamJaeiilaWIaeiOla4IaeiOla4IaeiOla4IaeGOmaiZaaWbaaSqabeaaieaacqGFYbGCcqGHsislcqaIYaGmaaGccqGHsislcqaIXaqmaeaacqGHuoarcqWGxbWvdaqhaaWcbaGaeGOmaiJaemiCaaNaey4kaSIaeGymaedabaGaemOCaihaaOGaeyypa0ZaaSaaaeaacqaIXaqmaeaacqaIYaGmaaGaeyiLdqKaem4vaC1aa0baaSqaaiabdchaWbqaaiabdkhaYjabgkHiTiabigdaXaaakiabgkHiTiab=n7aNnaaDaaaleaacqWGWbaCaeaacqWGYbGCaaaakeaacqWGYbGCcqGH9aqpcqaIYaGmcqGGSaalcqaIZaWmcqGGSaalcqGGUaGlcqGGUaGlcqGGUaGlcqGGSaalcqGFsbGuaeaacqWFZoWzdaqhaaWcbaGaemiCaahabaGaemOCaihaaOGaeyypa0JaemOta40aaeWaaeaacqaIWaamcqGGSaalcqaIYaGmdaahaaWcbeqaaiabgkHiTiabdkhaYbaaaOGaayjkaiaawMcaaaqaaaaacqGGSaalcaWLjaGaaCzcamaabmaabaGaeGymaeJaeGOmaidacaGLOaGaayzkaaaaaa@8CC9@

where N(*m*, *σ*^2^) is a Gaussian random number with mean *m *and standard deviation *σ*.

In this way, previously generated Wiener increments are always reused and new intermediate Wiener increments are always conditioned on previously generated ones. In addition, realizations of the two dimensional Itô integrals for a particular time increment must never be generated twice. The values of the Wiener increments, the current time step, and the two dimensional Itô integrals are then inputted into either the Euler-Maruyama or the Milstein numerical integrators and a trajectory of the system's dynamics for the next time step is obtained.

### The graphical user interface

**Figure 1:**
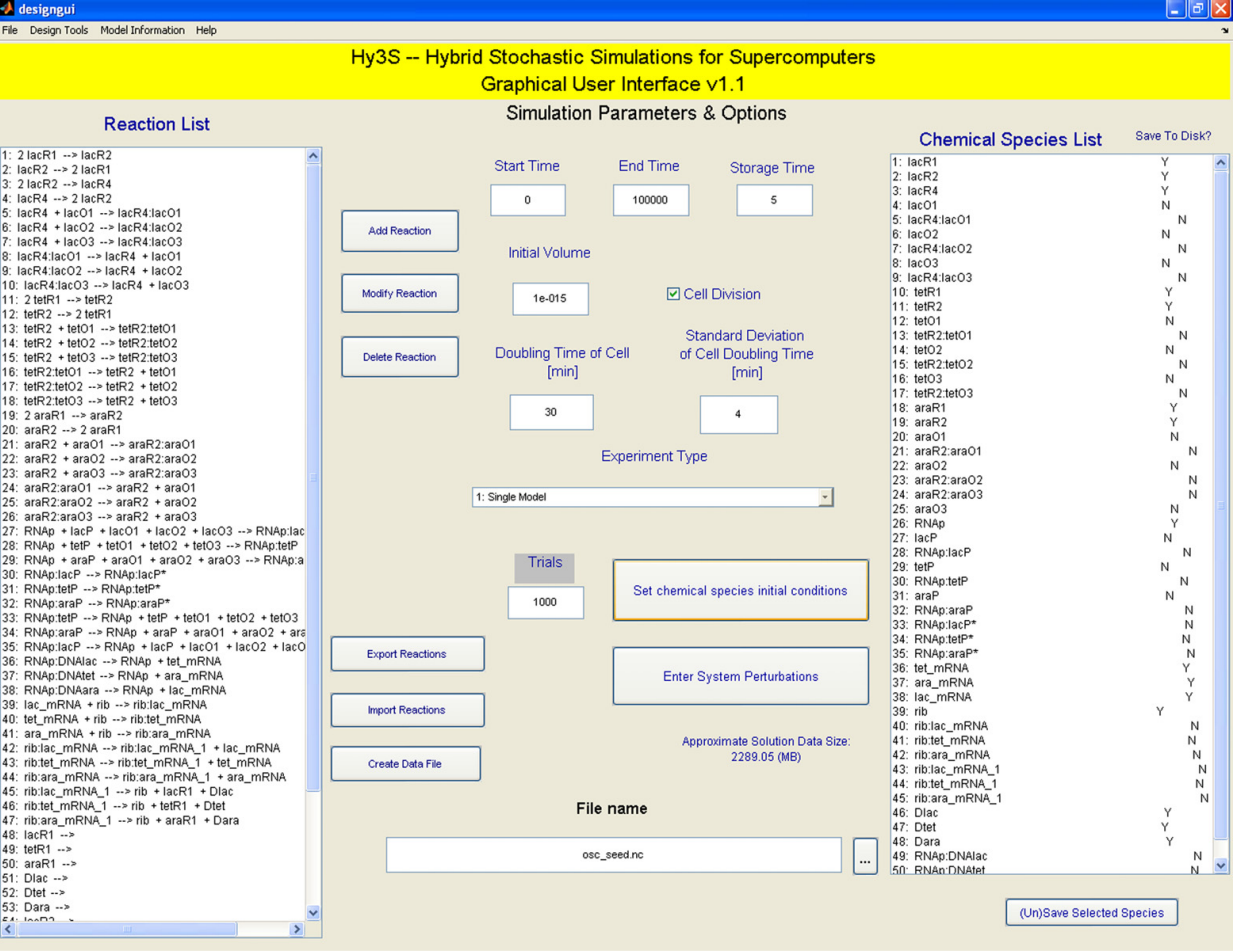
The main window of the graphical user interface.

**Figure 2:**
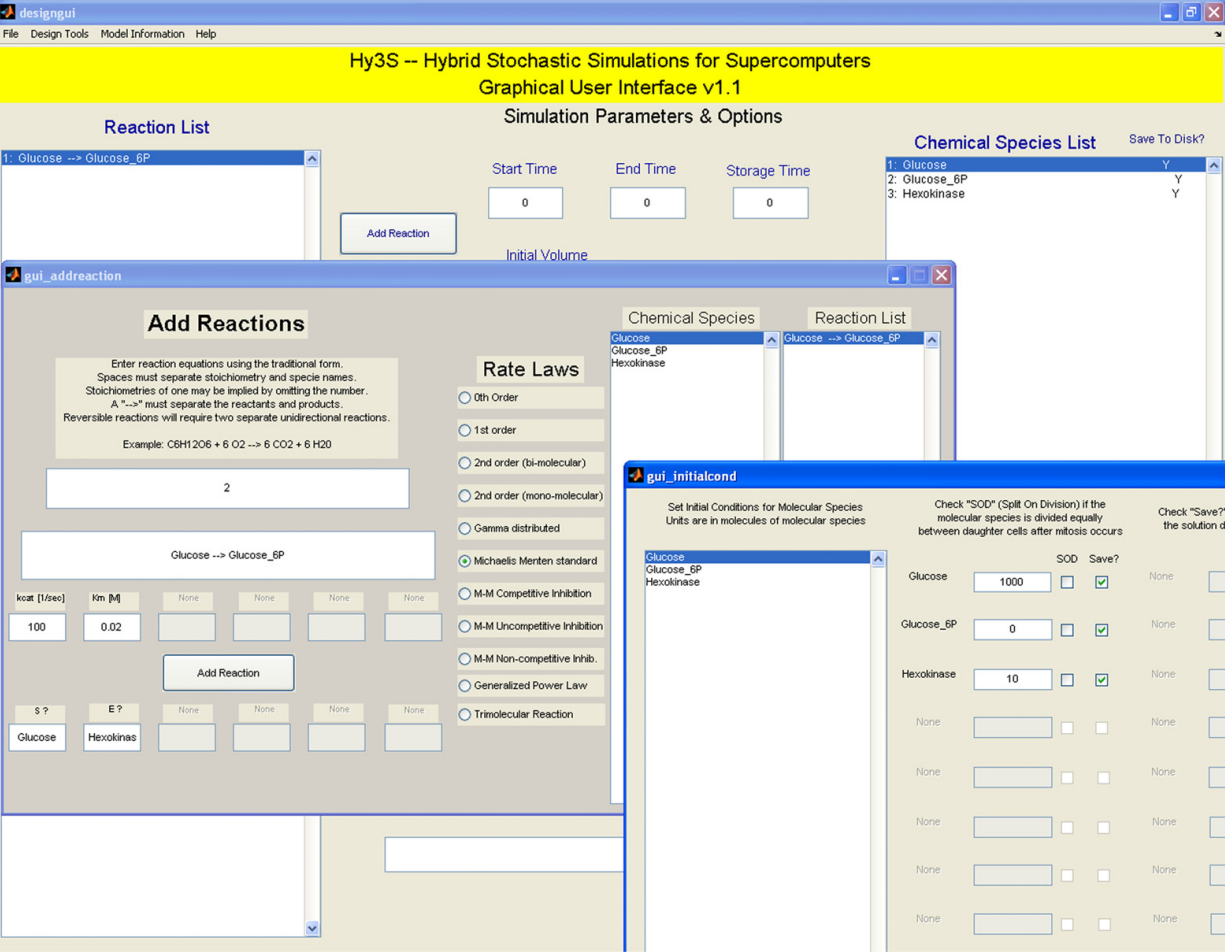
Two auxiliary windows showing the interfaces for adding reactions and setting initial conditions.

**Figure 3:**
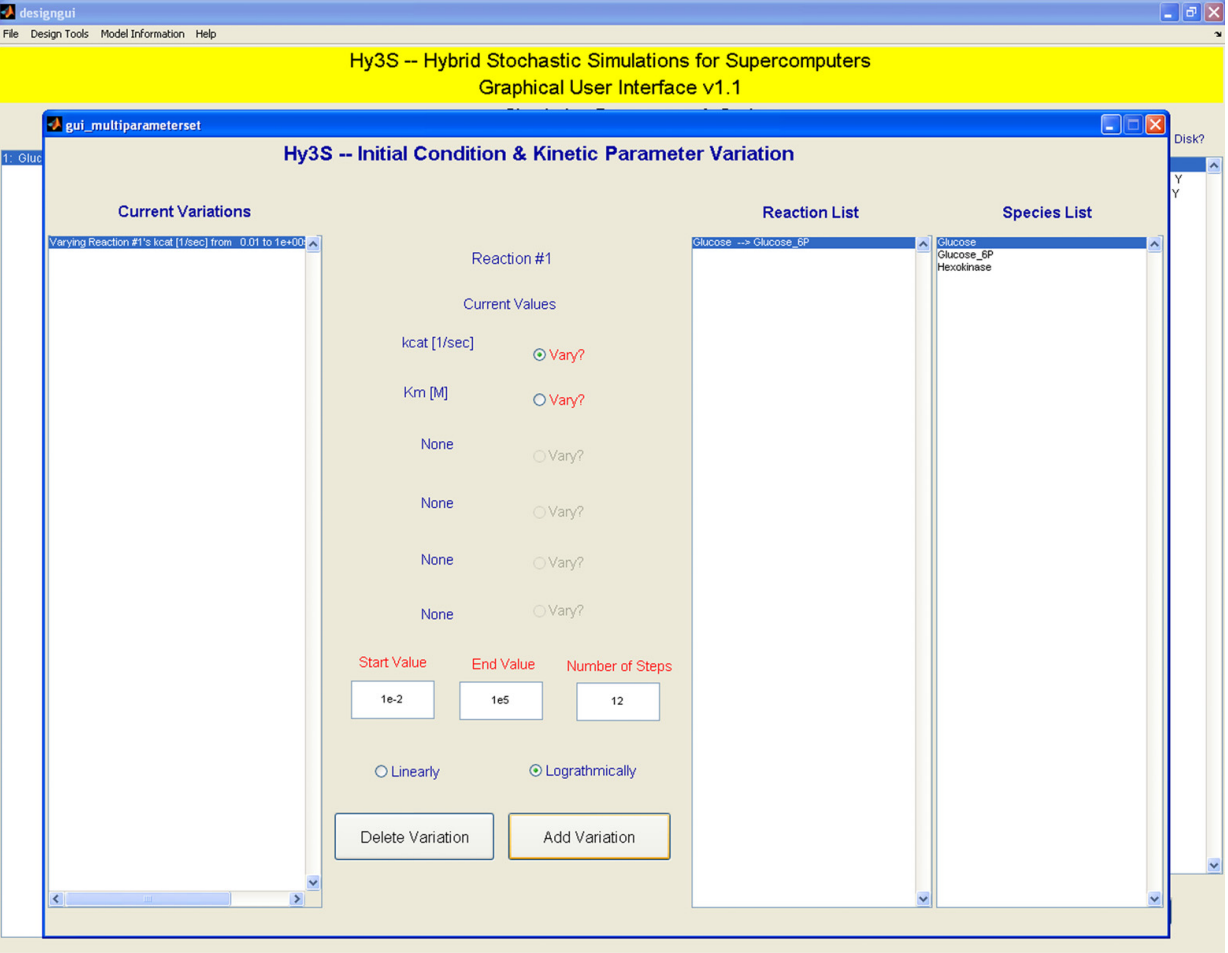
An auxiliary window showing the interface for adding systematic variations of kinetic parameters or initial conditions

The graphical user interface allows users to quickly create biochemical networks, set the necessary parameters and model data, and create the input NetCDF file. We use the open source project MexCDF [33] to create and compose NetCDF files from within Matlab. The graphical interface consists of one main window (Fig 1) with multiple auxiliary windows. Two of the auxiliary windows are shown in Figures 2 and 3. The model data includes the system of chemical or biochemical reactions, the initial conditions of species, the initial volume, the start and end times, the number of save time points, and the number of independent trials. Users may also add special discrete events, including cell replication events, gamma-distributed reactions, and timed perturbations to both the numbers of molecules of any chemical species or the kinetic parameters of any reaction. Finally, to help perform a simple sensitivity analysis, users may also create multiple models within a single NetCDF file, where each model has a different set of initial conditions or kinetic parameters of reactions.

#### Adding reactions and setting initial conditions

For each reaction, the user enters the stoichiometry of the reactant and product species, the rate law, and the kinetic parameters (see Fig 2). There are eleven different rate laws currently available and instructions for adding more are included. Besides the commonly used mass action rate laws, we included other rate laws that have been shown to be useful, such as generalized power law kinetics [34], special gamma-distributed events [13], and Michaelis Menten (M-M) type rate laws [35]. To make the process of adding reactions more efficient, the GUI will assume mass action kinetics and fill in the appropriate information in the species boxes (Fig 2, below the 'Add Reaction' button). The user can then enter in the kinetic parameters for the reaction or select an alternate rate law from the list and fill in the necessary information. By separating the stoichiometry of the reaction from its rate law, the software gives the user increased flexibility in adding a wide variety of reactions. The initial conditions for each chemical species are entered in units of molecules. Species may be selected to 'Split On Division' (SOD) so that, with every cell replication, the number of molecules of the selected species are distributed to two daughter cells. Because one is typically not interested in the stochastic dynamics of every species in a model, the software also allows users to discard or save the solution data of each species.

#### Adding special events

Biological systems often exhibit behaviours that are not easily modeled by a system of biochemical reactions. To aid in better simulating such behaviour, we include multiple types of special events, each describing a specific biological process. While sometimes ignored or approximated as a continuous rate of dilution, cell replication is more accurately modelled as a discrete event that disperses soluble molecules to daughter cells. The times at which cells divide are not constant, but typically fluctuate around an average with a Gaussian distribution. On the main GUI window, users may enable cell division special events and enter the mean and standard deviation of the cell replication times (Fig 1). The volume of the cell, starting from the initial value, increases exponentially with a rate equal to the inverse of the mean cell replication time and is reset to the initial volume when cell replication occurs.

Transcriptional and translation elongation is another process that is typically ignored, but may significantly affect the qualitative dynamics of the system through the introduction of both a delay in mRNA and protein production and an increase in stochasticity. One may describe the movement of the RNA polymerase or ribosome inchworming across the DNA or mRNA as a system of N first order reactions, where N is the number of base pairs or codons. However, because N is typically very large and decreases the efficiency of the simulation, one can assume that the rate of elongation, k, is constant and derive a single gamma-distributed event for the entire process of elongation [13]. When adding reactions, users may elect to describe the transcriptional and translation elongation events as gamma-distributed with rate k and N steps, providing an effective balance between accuracy and efficiency.

It is often convenient to modify the kinetic parameters of a reaction or the number of molecules of a chemical species mid-way through a simulation in order to test the system's response to an external perturbation or model some complex phenomenon. For example, one can model the addition of an inducer at some time by either increasing the number of molecules or increasing the rate of influx of the inducer midway through the simulation. One can also increase the influx of a receptor-binding ligand at some time to determine the response characteristics of a signal transduction network. Multiple perturbations to the system can model complex external behaviours. System perturbations are added in an auxiliary window, reachable from the main window.

#### Specifying multi-model NetCDF files and simulations

While studying a natural biological system or designing a synthetic one, scientists and engineers would often like to vary a kinetic parameter or initial condition of the biological model and determine its effect on the dynamics of the system. Instead of constructing numerous separate NetCDF files, each containing one set of model and solution data, Hy3S allows users to create a multi-model NetCDF file. A multi-model NetCDF file may contain multiple different biological models, each containing different kinetic parameters of reactions or initial conditions. If a simulation program is given a multi-model NetCDF file it will simulate the stochastic dynamics of each model, including the specified number of independent trials, and place the solution data back in the NetCDF file. The solution data is then four-dimensional (Number of Models × Number of Trials × Number of Timepoints × Number of Saved Species) and can be wholly or partially read into a data analysis program, such as Matlab. One can then perform a simple sensitivity analysis of a biological model by varying one or more parameters, simulating the stochastic dynamics of each model, and analyzing the dynamics as a function of a parameter.

In order to specify a multi-model NetCDF file, the user may select an Experiment Type of 2 or 'Combinatorial variation of kinetic parameters and initial conditions' and add variations by pressing the newly named button below the drop down menu (Fig 3). Kinetic parameters or initial conditions may be varied from a start to end value with any number of either linear or logarithmic steps. Adding two or more variations will cause the GUI to compute all combinations of each specified kinetic parameter and initial condition and place the appropriate information in the NetCDF file. Users may also construct an arbitrary list of kinetic parameters and initial conditions and place the information in the NetCDF file, which is helpful if one would like to vary one kinetic parameter and apply some constraint to others. For example, one may want to vary a backward kinetic constant while keeping the equilibrium constant unchanged. Creating multi-model NetCDF files helps to combine multiple models into a single, compact form, containing all of the model and solution data and enabling faster data analysis.

#### Creating complex biochemical networks and analyzing data with scripts

The graphical user interface provides a fast way to create small to medium sized biochemical networks. However, one would often like to create a very large network which has some special structure or characteristic. While one could enter in these reactions by hand, it is often more convenient to write a short program, or script, that constructs the biochemical network and places the necessary information in the NetCDF file. Because the NetCDF format is open and contains APIs in many different programming languages, many scientific applications may read and write NetCDF files. These scientific applications often contain miniature programming languages with many useful pre-built functions and enable users to program highly complex tasks in a small amount of time. We use the open source project MexCDF [29] to enable Matlab to read and write NetCDF files.

By combining the easy-to-use GUI with the capabilities of scripting, one can create any arbitrary biochemical network, possibly with thousands of reactions and chemical species, to answer a wide variety of research questions. Because of the variety of possible biochemical networks, it is not feasible to write a user interface to include everything. Instead, users may use the GUI to begin constructing a biochemical network and use scripting to extend that network for any particular purpose. For example, one can examine the effects of gene dosage on the dynamics of a gene network by using the GUI to construct a single copy gene network and a script to duplicate all DNA sites and their corresponding reactions to a specified copy number. One can also use scripting to quickly construct signal transduction networks which often have a combinatorially large number of chemical species due to numerous protein-protein interactions. We include a few example Matlab scripts that read and write Hy3S NetCDF files to help users write their own.

The obstacles to simulating large, realistic biological systems include not only construction and simulation of the model, but also the analysis of the solution data. Because the solution data set can be large, especially for multi-model NetCDF files, it is often necessary to read in only a portion of the solution at a time. Not all file formats allow one to read in a subset of data. However, NetCDF is specifically optimized for direct access mode. One can easily read or write hyperslabs of data using a NetCDF API, such as MexCDF in Matlab. For example, if one wants to compute a probability distribution of a particular species at a single time point then only a very small subset of the data needs to be read, which is easily performed and saves a tremendous amount of time. In addition, when constructing multi-model NetCDF files, one can extract the solution data along the model dimension, allowing for the easy analysis of the effects of a kinetic parameter or initial condition. Combining the optimized NetCDF format, the MexCDF interface, and Matlab functions, one can quickly analyze solution data using a variety of techniques, create figures, and achieve high research productivity.

## Results

We present three examples to examine different characteristics of Hy3S. The first example is a simple one that thoroughly analyzes the accuracy of the HyJCMSS numerical method. The second example is a large scale system benchmark with up to twenty thousand reactions that explores Hy3S's ability to simulate large biochemical networks. The third example, a bistable multiscale biochemical network with spontaneous escape, demonstrates Hy3S ability to simulate complex, realistic networks. All reported computational times are from simulations run on Itanium2 1.5 Ghz processors.

### An extensive test of accuracy

When we first proposed the numerical method simulating a hybrid jump/continuous Markov process [26] we analyzed the method's accuracy with numerous examples, including a simple reaction network consisting of a linear three-cycle of fast/continuous reactions and two non-linear slow reactions, named the 'Cycle Test'. However, we would like to demonstrate that the method's accuracy is not limited to linear fast/continuous reactions. Here, we use a 'Non-linear Cycle Test', containing three fast/continuous and two slow reactions (described in Table 3) and perform a series of accuracy measurements to determine how the error in the probability distribution, mean, and variance of the solution changes with the system size. The system size is a parameter in the reaction network that describes how well the fast/continuous reactions may be validly approximated as a continuous Markov process. Here, we take the system size to be the number of initial reactant and product molecules in the fast/continuous reactions.

**Table 3:** A Non-Linear Cycle Test

Fast/Continuous Reactions		Slow Reactions	
A + B → C + F	k = 1/2 kf	E + B → G	k = k_s_

C + D → E + B	k = k_f_	A + D → H	k = 1/2 k_s_
E + F → D + A	k = 3/2 k_f_		
k_f _= 1505500 [M sec]^-1^		k_s _= 1.0 Na V/Ω^2 ^[M sec]^-1^	
Ω = {100, 200, 316, 1000, 3162 10 000} [Molecules]		V = 1e-15 [Liters] T = [0, 10] [seconds]	
#A_o _= #B_o _= #C_o _= #D_o _= #E_o _= #F_o _= Ω, #G_o _= #H_o _= 0 [Molecules]			

The hybrid jump/continuous Markov stochastic simulator has two sources of error. The first is the approximation of the fast/continuous reactions as a continuous Markov process. The second is the numerical integration of the chemical Langevin and differential Jump equations. As the system size is increased, we expect that the first component of the error to decrease. As we decrease the time step of numerical integration, the second component of the error should also decrease. The magnitude of the second error is proportional to Δt^*γ*^, where *γ *is either the strong or weak order of accuracy of the stochastic numerical integration method, depending on the definition of the error. By varying the time step of numerical integration, we can measure the contribution of the second error source.

Ideally, to compare the HyJCMSS method using either the Euler-Maruyama or Milstein schemes, one should compute the strong error of the solution by comparing the differences in the *trajectories *between the hybrid approximate and exact solutions. To compute the strong error of the numerical solution of a system of stochastic differential equations (SDEs), one fixes the Brownian paths of the system and compares the evaluation of the solution using either different numerical schemes or, if available, an exact analytical solution. However, because we are simulating a coupled jump/continuous Markov process, it is more difficult to 'fix' the random process and evaluate the strong error. Consequently, we will only compute the weak mean and variance errors and, for easier comparisons, normalize them with respect to the exact mean and variance, so that

Δmeani(t)=|E[XiSSA(t)]−E[XiHy3S(t)]|E[XiSSA(t)]Δvar⁡i(t)=|var⁡{XiSSA(t)}−var⁡{XiHy3S(t)}|var⁡{XiSSA(t)}.     (13)
 MathType@MTEF@5@5@+=feaafiart1ev1aaatCvAUfKttLearuWrP9MDH5MBPbIqV92AaeXatLxBI9gBaebbnrfifHhDYfgasaacH8akY=wiFfYdH8Gipec8Eeeu0xXdbba9frFj0=OqFfea0dXdd9vqai=hGuQ8kuc9pgc9s8qqaq=dirpe0xb9q8qiLsFr0=vr0=vr0dc8meaabaqaciaacaGaaeqabaqabeGadaaakeaafaqabeGabaaabaGaeyiLdq0aa0baaSqaaiabd2gaTjabdwgaLjabdggaHjabd6gaUbqaaiabdMgaPbaakiabcIcaOiabdsha0jabcMcaPiabg2da9maalaaabaWaaqWaaeaacqWGfbqrdaWadaqaaiabdIfaynaaDaaaleaacqWGPbqAaeaacqWGtbWucqWGtbWucqWGbbqqaaGccqGGOaakcqWG0baDcqGGPaqkaiaawUfacaGLDbaacqGHsislcqWGfbqrdaWadaqaaiabdIfaynaaDaaaleaacqWGPbqAaeaacqWGibascqWG5bqEcqaIZaWmcqWGtbWuaaGccqGGOaakcqWG0baDcqGGPaqkaiaawUfacaGLDbaaaiaawEa7caGLiWoaaeaacqWGfbqrdaWadaqaaiabdIfaynaaDaaaleaacqWGPbqAaeaacqWGtbWucqWGtbWucqWGbbqqaaGccqGGOaakcqWG0baDcqGGPaqkaiaawUfacaGLDbaaaaaabaGaeyiLdq0aa0baaSqaaiGbcAha2jabcggaHjabckhaYbqaaiabdMgaPbaakiabcIcaOiabdsha0jabcMcaPiabg2da9maalaaabaWaaqWaaeaacyGG2bGDcqGGHbqycqGGYbGCdaGadaqaaiabdIfaynaaDaaaleaacqWGPbqAaeaacqWGtbWucqWGtbWucqWGbbqqaaGccqGGOaakcqWG0baDcqGGPaqkaiaawUhacaGL9baacqGHsislcyGG2bGDcqGGHbqycqGGYbGCdaGadaqaaiabdIfaynaaDaaaleaacqWGPbqAaeaacqWGibascqWG5bqEcqaIZaWmcqWGtbWuaaGccqGGOaakcqWG0baDcqGGPaqkaiaawUhacaGL9baaaiaawEa7caGLiWoaaeaacyGG2bGDcqGGHbqycqGGYbGCdaGadaqaaiabdIfaynaaDaaaleaacqWGPbqAaeaacqWGtbWucqWGtbWucqWGbbqqaaGccqGGOaakcqWG0baDcqGGPaqkaiaawUhacaGL9baaaaaaaiabc6caUiaaxMaacaWLjaWaaeWaaeaacqaIXaqmcqaIZaWmaiaawIcacaGLPaaaaaa@A92E@

Both the hybrid approximate and the exact mean and variances are computed by running at least 10 000 independent trajectories of the system. By using the weak definition of error, we should find that both the Euler-Maruyama and the Milstein numerical schemes have an order of accuracy of 1.0. We also compute the probability distributions of the exact and hybrid approximate solutions and the average, normalized weak mean and variance errors, where the average is taken over all time points and species.

**Figure 4:**
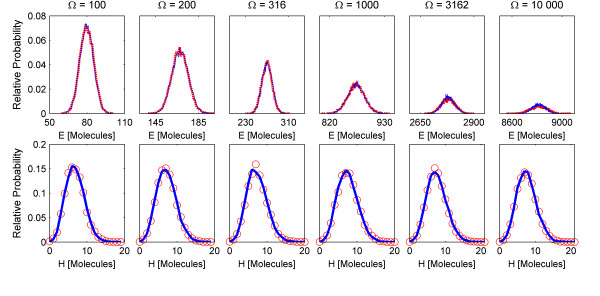
**Probability Distributions of the Non-Linear Cycle Test. **Probability distributions of species (Top) E and (Bottom) H of the Non-Linear Cycle Test at a time of 10 seconds for increasing system sizes, ranging from 100 to 10 000. The chemical Langevin and differential Jump equations are integrated using a fixed Euler-Maruyama scheme with a time step of 0.01 seconds. All other parameters are set to default values.

For all of the following simulations, we use the default values of the HyJCMSS parameters, which are (*ε*, *λ*, MSR Tol) = (100, 10, 0.01), where MSR Tol is the maximum tolerance for the Multiple Slow Reaction approximation. We begin by using the Euler-Maruyama scheme with a fixed time step of 0.01 seconds. As we increase the system size of the Non-Linear Cycle Test from 100 to 10 000, the ratio between the standard deviation and the mean of the solution goes to zero, indicating that we are effectively going towards the thermodynamic limit. The probability distribution of the solution is accurately captured *for all system sizes *and for *both *types of species, ones which are and are not affected by fast/continuous reactions (Fig 4). At a system size of 100, the HyJCMSS method treats at least two of the putative fast/continuous reactions as slow because the numbers of reactant or product molecules are so few. As the system size increases to 200 and larger values, all of the fast/continuous are, in fact, fast/continuous. Notice, however, that there is no noticeable difference in the solution between the system sizes of 100 and 200. The HyJCMSS method dynamically classifies reactions as fast/continuous, approximating them as a continuous Markov process only when it will produce an accurate solution. If there are no fast/continuous reactions, the method automatically reverts to the Next Reaction variant of the stochastic simulation algorithm. This combination of dynamic classification and algorithm switching makes the HyJCMSS method a *drop-in replacement *for the original stochastic simulation algorithm and its variants.

**Figure 5:**
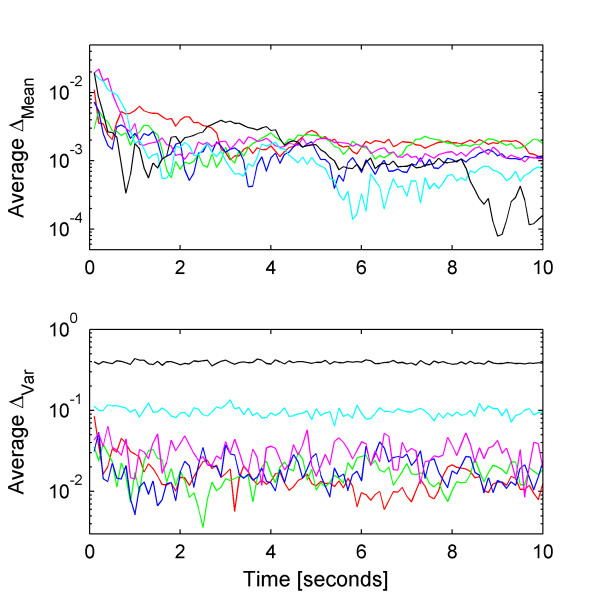
**Weak mean and variance errors of the Non-Linear Cycle Test. **The average normalized weak (Top) mean and (Bottom) variance errors of the Non-Linear Cycle Test using the Euler-Maruyama scheme with a fixed time step of 10^-2 ^seconds and system sizes of (red) 100, (green) 200, (blue) 316, (magenta) 1000, (cyan) 3160, and (black) 10 000. All other parameters are set to default values.

**Figure 6:**
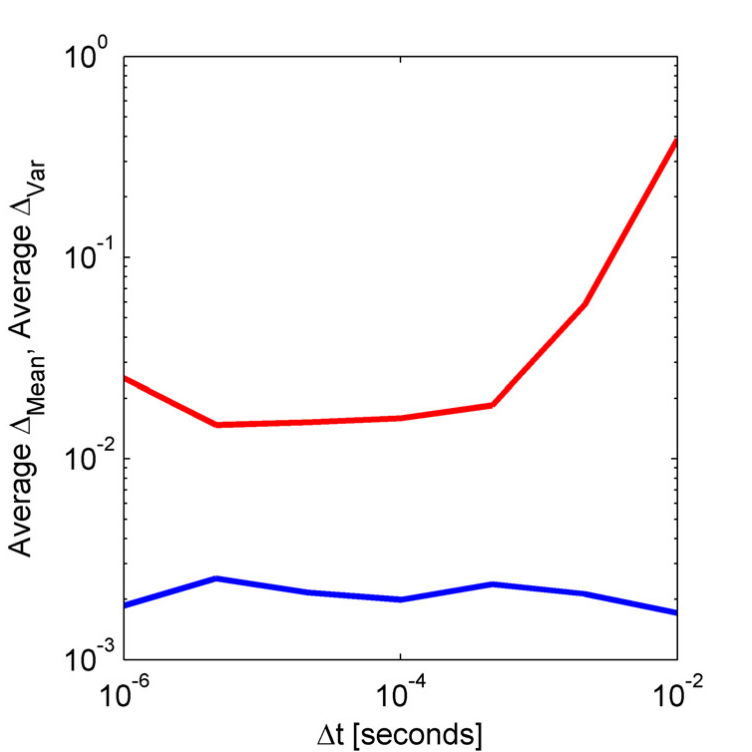
**Effect of integrator time step on weak mean and variance errors**The average normalized weak (blue) mean and (red) variance errors of the Non-Linear Cycle Test with a system size of 10 000 using the fixed time step Euler-Maruyama scheme with time step values ranging from 10^-6 ^to 10^-2 ^seconds. All other parameters are set to default values.

While increasing the system size, the stiffness of the chemical Langevin equation also increases. Stiffness is a measure of the disparity of timescales in a system of differential or other time-evolution equations. In Figure 5, we show the normalized weak mean and variance errors for increasing system sizes. By keeping the time step constant even as the system becomes more stiff, the second source of error increases, arising from the numerical integration of the SDEs. Notice that only the weak variance error increases with increasing system size while the error in the mean marginally decreases. This observation indicates that the stiffness originates from the terms containing the Wiener process, such as the diffusion term, and not the macroscopic terms, known as the drift. In order to obtain an accurate solution in terms of both the variance and the mean, we must use a time step that accounts for stiffness in either the drift-dominated or diffusion-dominated regimes. By decreasing the time step of numerical integration, we reduce the weak variance error with little change in the weak mean error (Fig 6).

**Figure 7:**
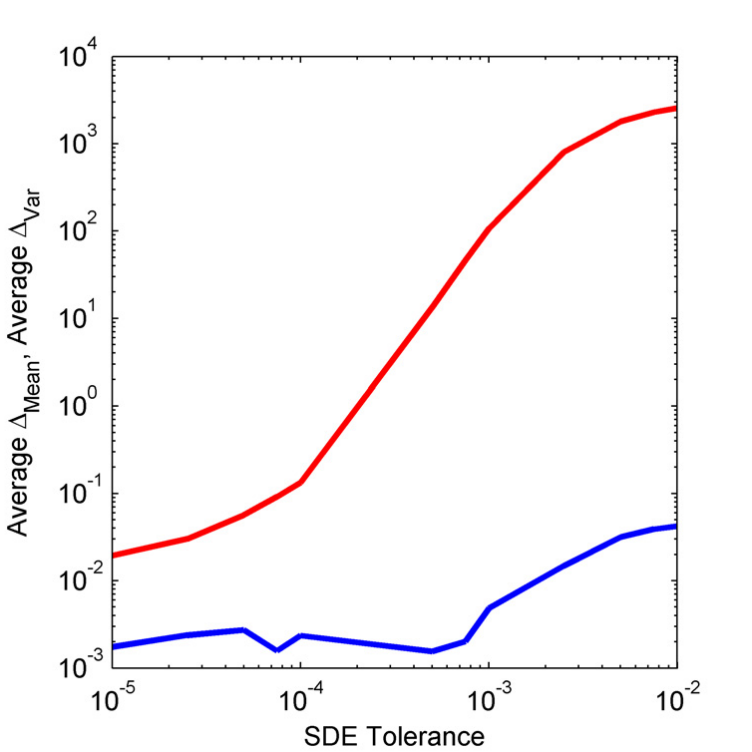
**Effect of adaptive scheme's user-defined tolerance on weak mean and variance errors. **The average normalized weak (blue) mean and (red) variance errors of the Non-Linear Cycle Test with a system size of 10 000 using the adaptive time step Milstein scheme with user-defined SDE tolerance values ranging from 10^-2 ^to 10^-5^. All other parameters are set to default values.

It would be highly useful to automatically and dynamically determine a time  step that produces only a small amount of numerical error, in terms of both  the weak mean and variance. Here, we use our implementation of the adaptive  Milstein method to examine how the user-defined tolerance affects the  accuracy of the solution of the Non-Linear Cycle Test. Using a system size  of 10 000, we vary the user-defined tolerance from 10^-5^ to 10^-2^ , showing  the weak mean and variance errors in Figure 7. While  alleviating the user from determining an accurate time step, adaptive time  stepping schemes have a higher overhead than fixed step ones and may require  more computational time. However, if the system only exhibits transient or  intermittent stiffness, then an adaptive time stepping scheme may be more  computationally efficient by using a smaller time step when the system is  stiff and a larger one when it is not.   Further work in this area is anticipated.

One important characteristic of the HyJCMSS method is that it converts the solution of a hybrid system governed by both Master and Fokker-Planck equations into the solution of a system of stochastic differential equations. The theory behind the numerical solution of SDEs has developed well enough that one can describe the asymptotic convergence properties, leading orders of accuracy, and numerical efficiency of a variety of numerical schemes that solve a large class of Wiener process driven SDEs, including the chemical Langevin and differential Jump equations. By testing the accuracy of the method on a small, but fully non-linear, example, we can demonstrate that the method's accuracy is fully governed by the numerical integration of the SDEs and that the accuracy may be controlled with a small number of user-defined parameters, such as the time step of numerical integration or the maximum tolerance of the local error.

### Benchmarks

The Non-Linear Cycle Test is a toy system that demonstrates the accuracy of the HyJCMSS method. However, realistic systems contain many *thousands *of reactions and chemical species. We use a large-scale system benchmark to test the computational efficiency of HyJCMSS when simulating large biochemical networks. The large-scale system benchmark is a system of R_f _bi-molecular 2^nd ^order fast/continuous reactions coupled to another set of R_s _bi-molecular 2^nd ^order slow reactions. The total number of reactions and chemical species are, respectively, R_f _+ Rs and 3R_f _+ 2 R_s_. The minimum degree of the dependency graph of the reaction propensities is always greater than ⌊R_s_/R_f_⌋ + 1, making this network less sparse than most biochemical networks. We increase R_f _and R_s _and measure the computational times of a simulation of a single trajectory using the HyJCMSS method with either the Euler-Maruyama or Milstein schemes with fixed time steps and also the Next Reaction variant of the stochastic simulation algorithm. Because the system is relatively non-stiff, we set the time step of numerical integration to 0.1 seconds. In Table 4, we show the computational times when varying both R_f _and R_s _from one to 10 000 reactions. The largest benchmark reaction network then has 20 000 reactions and 50 000 chemical species. It is clear that the HyJCMSS method is much faster than the stochastic simulation algorithm and that, to simulate large biochemical networks with only a few fast/continuous reactions, it is *highly necessary *to use a hybrid stochastic method.

**Table 4:** Comparison of computational times of a large-scale system benchmark

R_f_	R_s_	Fixed EM Time (seconds)	Fixed Milstein Time (seconds)	SSA Time (seconds)
2	1	0.0222	0.0632	5.870
2	100	0.5240	0.5792	54.07
2	1000	45.43	45.30	694.52
10	1000	44.78	44.39	3 481
100	1000	47.71	48.34	34 633
1000	1000	93.76	326.54	> 300 000
2	10000	3 394.1	3 970.9	12 159
100	10000	4 703.8	4 736.1	> 600 000
1000	10000	5 164.5	5 337.9	ND
10000	10000	13 099.0	30 601.5	ND

The computational times of a large-scale system benchmark using the fixed Euler-Maruyama (EM) and Milstein implementations of the HyJCMSS algorithm and the Next Reaction variant of the stochastic simulation algorithm (SSA). ND: Not Determined

### A complex bistable biochemical network with multiple timescales and spontaneous escape

Hy3S can also speed up the simulation of complex biochemical networks that exhibit interesting stochastic phenomena. To demonstrate its capabilities, we construct a hypothetical reaction network that features commonly found biological processes and generates behavior unique to random dynamical systems. The proposed biochemical network exhibits bistable behavior with spontaneous transitions between stable states and contains multiple timescales, including fast/continuous, fast/discrete, and slow/discrete reactions whose classification change during the simulation. The reactions in the network are extracted from three different types of biological processes: regulated gene expression, protein-protein binding interaction networks, and a hypersensitive enzymatic futile cycle commonly found in signal transduction cascades. This example shows that Hy3S is capable of accurately reproducing time-evolving multimodal distributions affected by disparate timescales, while consuming much less computational time. The biochemical reaction network consists of 49 reactions and 32 species and contains five coupled reaction modules:

• A protein-protein interaction network with monomers S1 through S4 each capable of binding to a protein scaffold, P. A scaffold bound to a monomer may also dimerize to another bound scaffold to form a four-count complex.

• The polymerization of S1:P_2_:S2 with itself to form both (S1:P_2_:S2)_2 _and (S1:P_2_:S2)_3_.

• A futile enzyme cycle consisting of two enzymes, E^f ^and E^b^, that rapidly deactivate and activate the scaffold, P.

• The Schlogl reaction network which generates bistability and spontaneous transitioning in the number of molecules of E^f^.

• The transcriptional and translational initiation and elongation of a gene which is activated by (S1:P_2_:S2)_3 _and produces the scaffold protein, P.

The reaction rate laws are all mass action kinetics with parameters set to physiologically possible values. The dynamical behaviour is qualitatively similar over a broad range of kinetic parameters. The full biochemical reaction network and its initial conditions and kinetic parameters are listed in Table 5.

**Table 5:** A bistable biochemical network with multiple timescales and spontaneous escape

Reactions	Kinetics	Reactions	Kinetics
S1 + P → S1:P	k_1_	∅→ E^f^	k_4_
S2 + P → S2:P	k_1_	E^f^→∅	k_5_
S3 + P → S3:P	k_1_	∅→ E^f^	k_6_
S4 + P → S4:P	k_1_	E^f^→∅	k_7_
S1:P + S2:P → S1:P_2_:S2	k_1_	E^f ^+ P → E^f^:P	k_8_
S1:P + S3:P → S1:P_2_:S3	k_1_	E^f^:P → E^f ^+ P	k_-8_
S1:P + S4:P → S1:P_2_:S4	k_1_	E^f^:P → E^f ^+ P*	k_8_^cat^
S2:P + S3:P → S2:P_2_:S3	k_1_	E^b ^+ P → E^b^:P	k_9_
S2:P + S4:P → S2:P_2_:S4	k_1_	E^b^:P → E^b ^+ P	k_-9_
S3:P + S4:P → S3:P_2_:S4	k_1_	E^b^:P → E^b ^+ P*	k_9_^cat^
S1:P → S1 + P	k_-1_	P:O + RNAP → P:O:RNAP	k_10_
S2:P → S2 + P	k_-1_	P:O:RNAP → P:O + RNAP	k_-10_
S3:P → S3 + P	k_-1_	P:O:RNAP → P:O + RNAP:DNA	k_11_
S4:P → S4 + P	k_-1_	P:O + (S1:P_2_:S2)_3_→ P:O*	k_12_
S1:P_2_:S2 → S1:P + S2:P	k_-1_	P:O* → P:O + (S1:P_2_:S2)_3_	k_-12_
S1:P_2_:S3 → S1:P + S3:P	k_-1_	P:O* + RNAP → P:O*:RNAP	k_13_
S1:P_2_:S4 → S1:P + S4:P	k_-1_	P:O*:RNAP → P:O* + RNAP	k_-13_
S2:P_2_:S3 → S2:P + S3:P	k_-1_	P:O*:RNAP → P:O* + RNAP:DNA	k_14_
S2:P_2_:S4 → S2:P + S4:P	k_-1_	RNAP:DNA → RNAP + mRNA	k_15_, N_15_
S3:P_2_:S4 → S3:P + S4:P	k_-1_	mRNA + Rib → Rib:mRNA	k_16_
2 S1:P_2_:S2 → (S1:P_2_:S2)_2_	k_2_	Rib:mRNA → mRNA+Rib:mRNA1	k_17_
S1:P_2_:S2 + (S1:P_2_:S2)_2_→ (S1:P_2_:S2)_3_	k_3_	Rib:mRNA1 → Rib + P	k_18_, N_18_
(S1:P_2_:S2)_2_→ 2 S1:P_2_:S2	k_-2_	mRNA →∅	k_19_
(S1:P_2_:S2)_3_→ S1:P_2_:S2 + (S1:P_2_:S2)_2_	k_-3_	P →∅	k_20_
Rate Laws & Kinetic Constants:			
0^th ^order: k_6 _= 200 [molecules/sec]			
1^st ^order: k_-1 _= 50, k_-2 _= k_-3 _= 0.2, k_7 _= 3.5, k_-8 _= k_-9 _= 1, k_8_^cat ^= 150, k_9_^cat ^= 50, k_-10 _= k_-13 _= 0.1, k_11 _= 0.01, k_-12 _= 1.155e-3, k_14 _= 0.2, k_17 _= 33, k_19 _= 2.31e-3, k_20 _= 1.1155e-3 [1/sec]			
2^nd ^order: k_1 _= 0.025, k_2 _= 1e-3, k_3 _= 1e-5, k_4 _= 0.015 (mono-molecular), k_8 _= 2.5e-4, k_9 _= 8.47e-5, k_10 _= 0.05, k_12 _= 0.01, k_13 _= 0.1, k_16 _= 5e-3 [molecules sec]^-1^			
3^rd ^order: k_5 _= 1e-4 (mono-molecular) [molecules^2 ^sec]^-1^			
Gamma-distributed events: k_15 _= 30 nt/sec, N_15 _= 1200 nt, k_18 _= 33 aa/sec, N_18 _= 400 aa			
Initial Conditions: #P_o _= #P* _o _= 4000, #S1_o _= #S2_o _= #S3_o _= #S4_o _= 1500, #Rib_o _= 300, #RNAP_o _= 180, #Ef_o _= #Eb_o _= 250, otherwise 0 [Molecules]			

The computational times of a large-scale system benchmark using the fixed Euler-Maruyama (EM) and Milstein implementations of the HyJCMSS algorithm and the Next Reaction variant of the stochastic simulation algorithm (SSA). ND: Not Determined

We simulate 10 000 trajectories of the biochemical network between 0 and 2000 seconds using the HyJCMSS algorithm with a fixed step Euler-Maruyama numerical integration method and a parameter set of (*ε*, *λ*, *MSR Tol*, Δt) = (30, 10, 0.10, 2e-3). The hybrid method requires only 82.0 seconds per trajectory while the Next Reaction variant of the stochastic simulation algorithm requires 9980.6 seconds per trajectory for a total speed up of 121.7. Clearly, it would be highly impractical to run 10 000 trajectories of this system with the original method. By using Hy3S, one can obtain the entire time-evolving probability distribution of the system in about 227 cpu hours. By using multiple processors, the actual time to produce the solution may be reduced by up to 10 000 fold.

**Figure 8:**
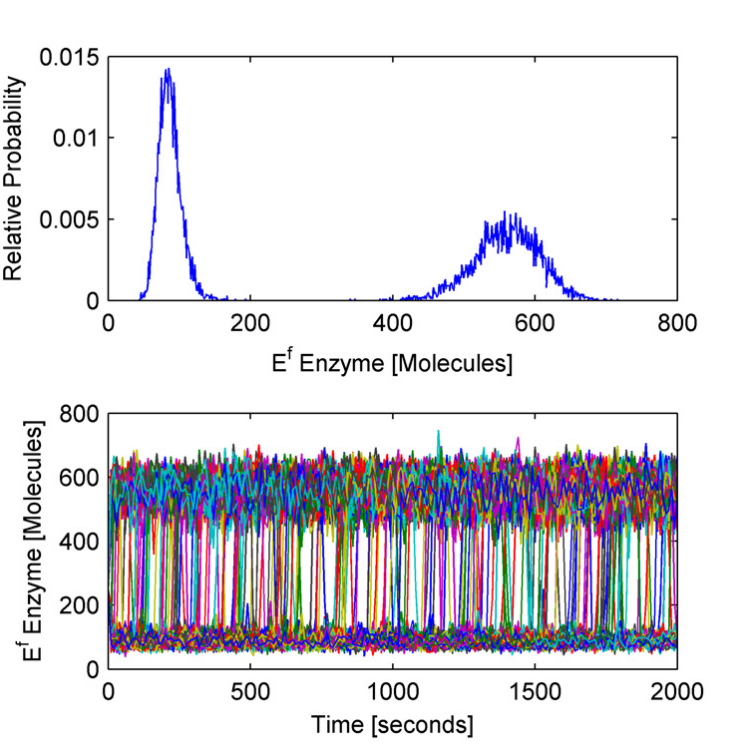
**Distribution and trajectories of the Schlogl reaction module**(Top) The relative probability distribution of the number of E^f ^molecules at 50 seconds. (Bottom) Out of 10 000 independent trajectories, the 225 shown here exhibit spontaneous transitions from low to high or high to low numbers of E^f ^molecules.

**Figure 9:**
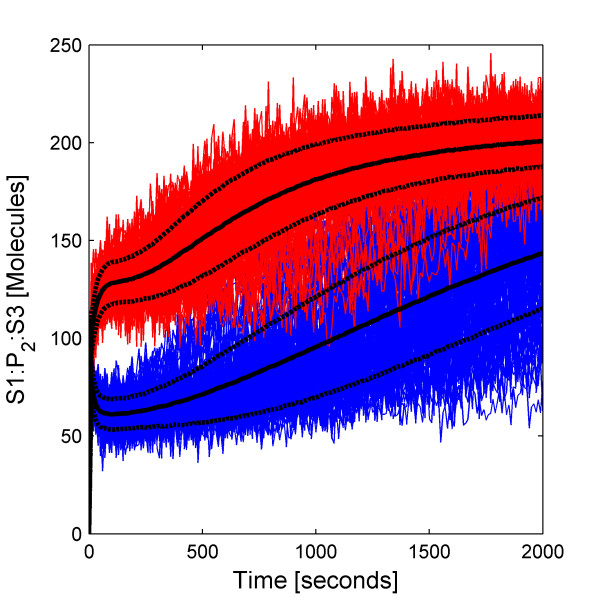
**Branching of solution affects bound scaffold complexes. **An ensemble of 10 000 trajectories of the S1:P_2_:S3 scaffold complex, where trajectories are colored according to the branch of the solution. The number of E^f ^molecules resides in either the (red) low or (blue) high stable state. Both the (black solid lines) mean and (black dashed lines) mean ± standard deviation are shown for both branches.

**Figure 10:**
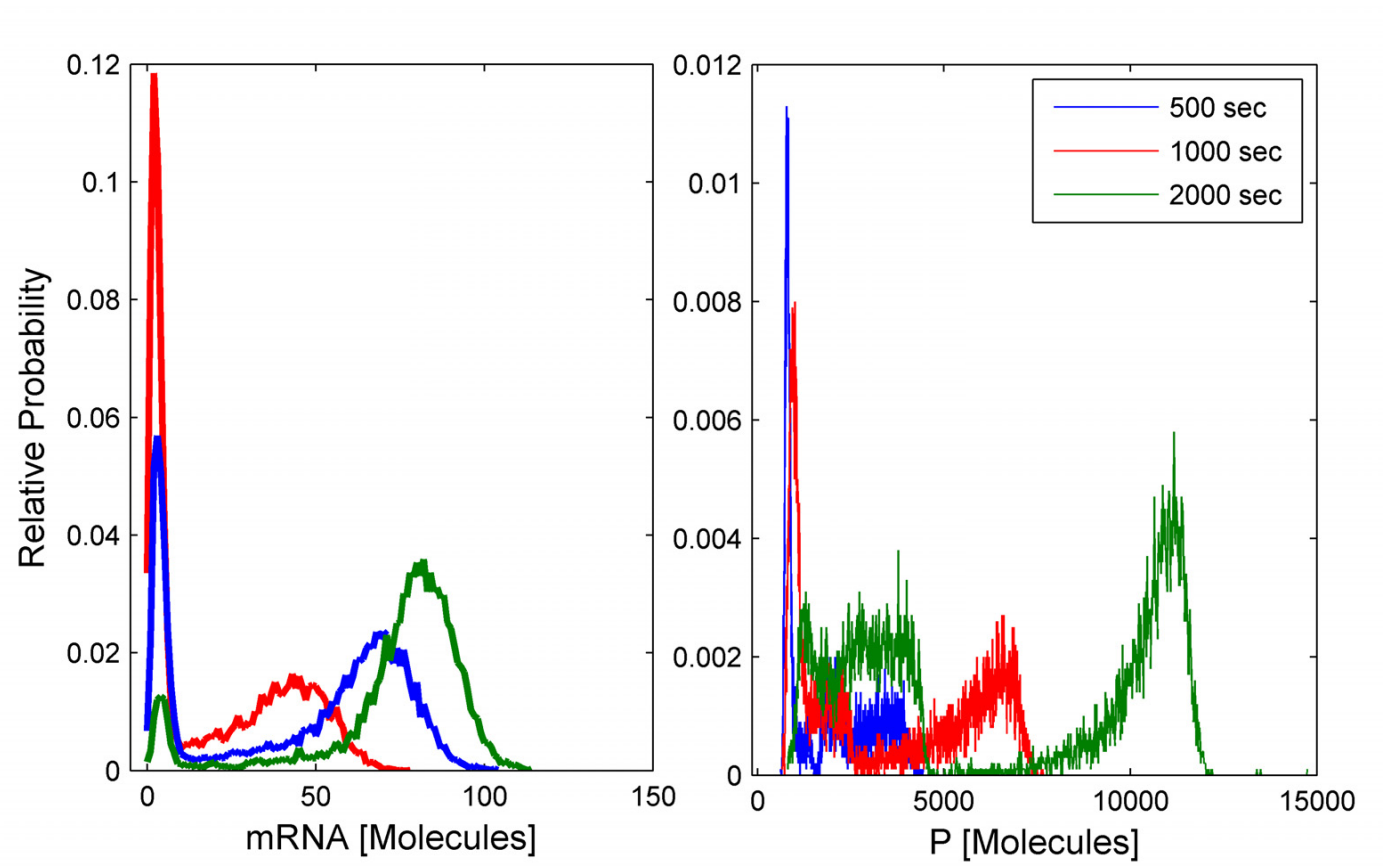
**Multimodal distributions of mRNA and scaffold molecules over time. **The relative probability distribution of the numbers of (Left) mRNA molecules and (Right) free scaffold molecules at (blue) 500 seconds, (red) 1000 seconds, and (green) 2000 seconds.

**Figure 11:**
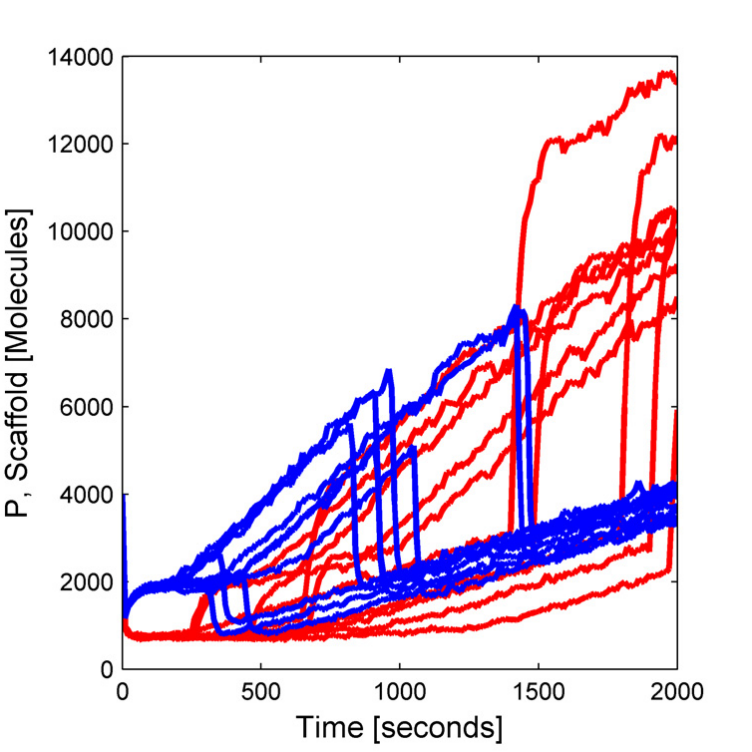
**Spontaneous escape results in rapid switching between branches of solution. **The effect of spontaneous escape in the numbers of E^f ^molecules on the number of free scaffold complexes, P. Trajectories are colored according to their final solution branch, where the numbers of E^f ^molecules transitions to either the (blue) high or (red) low stable states. Only 20 representative trajectories are shown.

The dynamics of the biochemical network are both interesting and complex. Starting from a single initial condition, the Schlogl reaction module causes the number of molecules of enzyme E^f ^to rapidly converge to a bimodal distribution (Fig 8, top) with peaks at around 80 and 575 molecules and also to spontaneously transition from the low to high stable states and vice versa (Fig 8, bottom) with a specific rate of about 1.1e-5 sec^-1 ^for either transition. While E^f ^fluctuates within its low stable state, the number of active scaffold proteins is high, resulting in larger numbers of bound scaffold complexes, complexed dimers, and also complexed trimers (Fig 9). While E^f ^fluctuates within its high stable state, the number of active scaffold proteins is much fewer and all scaffold-containing complexes are reduced in number. Because the complexed trimer, (S1:P_2_:S2)_3_, activates transcriptional initiation, the expression of the gene is also bistable, including the numbers of mRNA transcripts and active scaffold protein (Fig 10). The expression of the gene produces more active scaffold protein which results in greater numbers of scaffold-containing complexes, including the activator, and causing a general positive feedback loop. A modest amount of leaky gene expression counters some of the bistability in gene expression. Trajectories of E^f ^which undergo a spontaneous transition between stable peaks will quickly and dramatically change the number of active scaffold proteins (Fig 11). The main point of this example is that Hy3S enables the simulation of highly complex biochemical networks in a practical amount of computational time. A realistic biochemical network will be at least as complex as this example and will contain multiple disparate timescales. Hy3S can simulate these systems whereas, in a practical amount of time, the exact stochastic simulation methods can not.

## Discussion

Hy3S, or Hybrid stochastic simulation for supercomputers, enables scientists and engineers to simulate the stochastic dynamics of arbitrary homogeneous chemical and biochemical reaction networks. These models can accurately capture the intracellular dynamics of biological organisms at the single-cell level and may be used to both study natural biological systems and design synthetic ones. We have implemented our recently developed hybrid jump/continuous stochastic simulation method [26] (HyJCMSS) into a fully functional, robust software package, capable of simulating models of biological systems with thousands of fast/continuous or slow reactions and chemical species. We use four different stochastic numerical integrators with either fixed or adaptive time stepping and with different orders of strong accuracy. The simulation programs utilize optimizing data structures and are parallelized using MPI, enabling efficient simulations on multiple processors. Multiple types of special events are included, such as cell replication and transcriptional and translation elongation, and additional ones are easily added. The simulation program code is open source and licensed under the Gnu General Public License (GPL). We have also created a simple, easy to use GUI that allows scientists and engineers to quickly create biochemical networks, add special events, and create multi-model simulations. The NetCDF data format is open, self-describing, and has APIs in numerous programming languages. The APIs can access the data in direct access mode, enabling fast read and write access to hyperslabs of data. NetCDF files may be read and written in popular scientific applications, such as Matlab, allowing complex biochemical networks to be constructed and solution data to be efficiently analyzed and plotted. Overall, Hy3S provides a high level of research productivity throughout the entire process of model composition, simulation, and analysis.

In the first public version of Hy3S, we have concentrated on simulating homogeneous biological systems with many fast/continuous and slow/discrete reactions. However, much of the theory behind the solution of a coupled jump/continuous Markov process may also be applied to heterogeneous systems. In addition, we and others have recently developed an 'equation-free' probabilistic steady state or partial equilibrium approximation that speeds up the simulation of *arbitrary *biochemical networks with 'fast/discrete' and 'slow/discrete' reactions [36-38]. A reaction is fast/discrete when it frequently occurs, but may not be validly approximated as a continuous Markov process. The method dynamically determines when the effects of a subset of fast/discrete reactions have converged to a quasi-stationary distribution, samples from the underlying distribution, and uses those samples to compute the time of the next slow/discrete reaction along with the state of the system at which it occurs. The method may be used in conjunction with HyJCMSS to treat homogeneous systems with many slow/discrete, fast/discrete, and fast/continuous reactions. Robust implementations of these and other advanced hybrid stochastic methods will be added to future versions of Hy3S.

### Open source possibilities

By releasing the source code of Hy3S under the GPL, we are offering the computational biology community the ability to copy, modify, and distribute the Hy3S source code with one main restriction: all distributed or published modifications of the source code must also contain the source code and follow the other restrictions of the GPL. We are releasing the code in this fashion for a few reasons. Most research projects have questions that a pre-built software package may not specifically answer. By modifying the source code to fit one's needs, one can tailor the program for a particular research project. We have designed the software to be as modular as possible to allow the easier reuse of code segments. For example, each simulation algorithm is encapsulated as a propagator, or a single subroutine that is given the current state of the system and returns the state of the system at some specified future time. One can greatly modify the program without touching the innards of the simulation algorithm. In addition, the simulation algorithms themselves are structured to allow the easy insertion of additional rate laws and special events. Instructions for adding additional special events and rate laws are included.

Perhaps the best reason for opening up the source code is that the field of stochastic chemical kinetics is still an immature one. New algorithms are frequently published and new techniques continue to be explored. By building on top of Hy3S, one can accelerate the pace of algorithm development and implementation while making newly developed methods useful to the general public. Newly developed algorithms can be fairly compared to current ones and the ones with the highest merit can be immediately used by the community. Participation and collaboration are always welcome. In this way, the software itself will quickly improve and mature to the highest standards.

## Conclusion

Hy3S allows scientists and engineers to compute the stochastic dynamics of large, realistic chemical or biochemical networks with *thousands *of reactions and chemical species. By using a recently developed hybrid jump/continuous Markov stochastic simulator (HyJCMSS), an accurate solution is obtained using much less computational time, as compared to the Next Reaction variant of the stochastic simulation algorithm. The software is also parallelized with MPI with near 100% efficiency, enabling high research productivity on relatively inexpensive Linux computing clusters, and contains numerous useful features to more accurately model biological systems.

## Availability and requirements

Project Name: Hy3S, Hybrid Stochastic Simulation for Supercomputers

Project Homepage: 

Operating System: Platform-independent (source code available)

Programming Languages: Fortran95

Other requirements: NetCDF v3.5.1 or higher, (For optional GUI: Matlab R14, MexCDF)

License: GNU GPL

Any restrictions on use by non-academics: None

## Authors' contributions

H.S wrote the manuscript and developed the initial source code. H.S and V.S expanded the source code to include the Milstein and adaptive time step schemes. Y.K conceived of the study, coordinated, and helped draft the manuscript. All authors read and approve of the final manuscript.

## Links


